# Efficacy and safety of the ayurvedic formulation ‘*Trikatu*’ as an add-on to standard care in dyslipidemia: Study protocol for a randomized, double-blind, placebo-controlled trial evaluating lipid parameters, and gut microbiota

**DOI:** 10.1371/journal.pone.0348058

**Published:** 2026-05-11

**Authors:** Shruti Khanduri, Sophia Jameela, Suchanda Sahu, Sujata Devi, Bhogavalli Chandra Sekhara Rao, Kshirod Ratha, Richa Singhal, Latika Kaushik, Narayanam Srikanth, Rabinarayan Acharya

**Affiliations:** 1 Department of Ayurveda, Central Council for Research in Ayurvedic Sciences, Ministry of Ayush, New Delhi, India; 2 Department of Biochemistry, All India Institute of Medical Sciences, Bhubaneswar, India; 3 Department of General Medicine, All India Institute of Medical Sciences, Bhubaneswar, India; 4 Department of Ayurveda, Central Ayurveda Research Institute, Bhubaneswar, India; 5 Scientist C, Indian Council for Medical Research-National Institute of Malaria Research, New Delhi, India; 6 Deputy Director General, Central Council for Research in Ayurvedic Sciences, Ministry of Ayush, New Delhi, India; 7 Former Director General, Central Council for Research in Ayurvedic Sciences, Ministry of Ayush, New Delhi, India; Guilan University of Medical Sciences, IRAN, ISLAMIC REPUBLIC OF

## Abstract

**Background:**

Dyslipidemia is a leading modifiable risk factor for cardiovascular disease (CVD), with early and sustained LDL-C reduction offering significant preventive benefits. However, in low- and middle-income countries, long-term adherence to statin therapy remains alarmingly low, highlighting the need for culturally acceptable and safer adjunctive options. *Trikatu*, a classical Ayurvedic formulation traditionally known to enhance metabolism, may offer supportive benefits in lipid regulation. This study protocol was developed to assess the efficacy and safety of *Trikatu* as an add-on to standard statin therapy on lipid parameters and assess changes in gut microbiota in response to the intervention and identify microbial correlates associated with favorable lipid outcomes.

**Methods:**

This randomized, double-blind, placebo-controlled clinical trial is being conducted at AIIMS Bhubaneswar, a tertiary care center. Total 170 participants (aged 25–60 years) with moderate to high Atherosclerotic cardiovascular disease (ASCVD) risk. Dyslipidemia, as defined by the 2019 ACC/AHA Guidelines and indicated for statin therapy, will be randomized in a 1:1 ratio to receive either *Trikatu* (1000 mg) or matching placebo, administered orally twice daily after food for 12 weeks along with standard statin therapy (dose/intensity as per guidelines). Primary outcome includes change in fasting serum LDL-C from baseline to 12 weeks. Secondary outcomes include changes in total cholesterol, HDL-C, triglycerides, glycemic markers (fasting glucose, HbA1c, insulin, HOMA-IR), inflammatory and metabolic markers (hs-CRP, TNF-α, IL-6, adiponectin, ghrelin, ApoA1, ApoB), resting blood pressure, and gut microbiota. The proportion of participants achieving normal cholesterol levels (<200 mg/dL) will also be assessed. Drug compliance and any adverse events will also be recorded.

**Discussion:**

In individuals with moderate to high ASCVD risk, achieving and sustaining optimal lipid control remains a cornerstone of cardiovascular prevention. However, poor long-term adherence to statin therapy, especially in low-resource settings, limits real-world impact. By assessing *Trikatu,* effects on lipid profiles, glycemic and inflammatory markers, and gut microbiota, the study aims to explore its potential to augment cardiovascular risk reduction in a holistic and culturally acceptable manner. If proven effective and safe, *Trikatu* could serve as a valuable complementary strategy in dyslipidemia management.

**Clinical Trial Registration:**

Clinical Trial Registry of India (CTRI/2023/04/051942) Registered on 25/04/2023.

## Introduction

Dyslipidemia, characterized by abnormal levels of serum cholesterol, triglycerides, and related lipoproteins, is a prevalent metabolic disorder with well-established links to atherosclerotic cardiovascular disease (ASCVD). Elevated LDL cholesterol plays a central role in the pathogenesis of atherosclerosis, and its reduction has been shown to significantly improve cardiovascular outcomes. Statins remain the cornerstone of dyslipidemia management; however, long-term adherence and statin tolerance remain suboptimal in many populations, particularly in low- and middle-income settings [1]. Large global studies such as INTERHEART and PURE have consistently highlighted key risk factors contributing to CAD, many of which are modifiable. These include lifestyle factors such as smoking, poor diet, physical inactivity, and in some populations, alcohol use and household air pollution. Metabolic risk factors like dyslipidemia (particularly abnormal ApoB/ApoA1 ratios or non-HDL cholesterol), hypertension, diabetes, and abdominal obesity are strongly associated with increased CAD risk. Social determinants, including psychosocial stress, depression, and low educational status, also significantly influence cardiovascular outcomes [2,3]. These findings emphasize the importance of a comprehensive and integrative approach to CAD prevention, addressing both biological and behavioral contributors—particularly lipid abnormalities, which remain among the most modifiable and impactful targets. The recent ICMR-INDIAB study (2023) highlights significant inter-state and regional disparities in the prevalence of hypercholesterolemia and other forms of dyslipidemia across India. Notably, the highest rates of elevated total cholesterol were observed in Kerala (50.3%), Goa (45.6%), and Himachal Pradesh (39.6%), whereas substantially lower prevalence was reported in Jharkhand (4.6%), Assam (7.9%), and Bihar (9.7%) [4].

Dyslipidemia is typically asymptomatic and often identified incidentally through blood tests. For primary prevention, nonpharmacological measures—including dietary modification, physical activity, and lifestyle changes—are the initial recommendation. However, in individuals at elevated cardiovascular (CV) risk, statins are the first-line pharmacologic therapy due to their proven efficacy in lowering low-density lipoprotein cholesterol (LDL-C). Clinical guidelines emphasize achieving LDL-C targets based on individual CV risk profiles, with statins forming the cornerstone of lipid-lowering strategies. Despite their benefits, statin trials have demonstrated persistent residual CV risk, prompting growing interest in addressing other lipid parameters, such as triglycerides (TG) and high-density lipoprotein cholesterol (HDL-C), to enhance overall cardiometabolic outcomes.

*Trikatu* is a classical Ayurvedic formulation composed of the powdered combination of three botanicals: *Zingiber officinale* (dry ginger rhizome), *Piper longum* (long pepper fruit), and *Piper nigrum* (black pepper fruit) [5]. Traditionally, it is widely prescribed as part of polyherbal formulations to enhance digestion and metabolic function. The active phytoconstituent piperine, present in both *P. longum* and *P. nigrum*, is known to enhance bioavailability and has demonstrated multiple pharmacological effects. Its principal bioactive compound, piperine, is known to enhance the bioavailability of co-administered drugs by reducing their elimination half-life and modifying pharmacokinetic parameters. Studies have highlighted *Trikatu’s* broad pharmacological profile, including anti-inflammatory, antioxidant, and immunomodulatory effects, mediated through suppression of pro-inflammatory cytokines such as tumor necrosis factor- α (TNF-α), interleukin-1β (IL-1β), inerleukin-6 (IL-6), and inerleukin-17 (IL-17) [6,7].

*Trikatu* has been considered as an adjunct to statin therapy in this clinical study on dyslipidemia to address residual lipid abnormalities and enhance therapeutic outcomes. While statins effectively lower LDL cholesterol, many patients continue to have elevated triglycerides and suboptimal HDL levels. *Trikatu*, composed of *Zingiber officinale*, *Piper longum*, and *Piper nigrum*, has shown potential to improve lipid profiles by reducing triglycerides and LDL [8]. Its bioenhancing property—primarily through piperine—can improve the bioavailability of statins, potentially optimizing efficacy at lower doses. Its anti-inflammatory and antioxidant effects may further contribute to cardiovascular risk reduction, making it a promising integrative adjunct in lipid management.

## Methods

### Objectives

The primary objective of this trial is to evaluate the efficacy and safety of the Ayurvedic formulation *Trikatu* as adjunct to statin in improving lipid parameters in individuals with dyslipidemia. The secondary objective is to assess the changes in gut microbiota composition in response to the intervention and to identify correlates predictive of favorable metabolic outcomes and also to assess the impact on metabolic and inflammatory biomarkers.

### Trial design

This study is a prospective, randomized, double-blind, placebo-controlled clinical trial. The intervention period will span 12 weeks, followed by a 4-week post-treatment observational phase to assess the sustainability of response. Eligible participants with borderline dyslipidemia will be randomly assigned in a 1:1 ratio to receive either *Trikatu* (TK) or a matching placebo. A total of five scheduled visits will be conducted: baseline (Visit 1, Day 0), interim assessments at Day 28 (Visit 2), Day 56 (Visit 3), and Day 84 (Visit 4), and a final follow-up visit without intervention on Day 112 (Visit 5).

### Trial setting

The clinical trial will be conducted at the Outpatient Department of General Medicine, All India Institute of Medical Sciences (AIIMS), Bhubaneswar—a tertiary care academic medical institution. All study-related procedures, including participant screening, intervention administration, clinical assessments, and laboratory investigations, will be conducted within the facility under the supervision of qualified investigators. The study will be carried out in compliance with the ethical principles outlined in the *Indian Council of Medical Research (ICMR) National Ethical Guidelines for Biomedical and Health Research Involving Human Participants, 2017*, and in accordance with the principles of the *Declaration of Helsinki* (latest revision, 2013).

### Eligibility criteria

#### Inclusion criteria.

Participants of either gender between 25–60 years of age, diagnosed with moderate to high ASCVD risk (10-year ASCVD risk ≥7.5%) dyslipidemia in accordance with the 2019 ACC/AHA Guidelines for the Primary Prevention of Cardiovascular Disease and indicated for statin therapy, will be considered eligible for inclusion. Eligible individuals must have a Body Mass Index (BMI) ranging from 18.5 to 34.9 kg/m², provide written informed consent after adequate understanding of study details, and express willingness to adhere to the intervention protocol for a 12-week duration.

#### Exclusion criteria.

Exclusion criteria include the use of lipid-modifying agents (other than statins), including Ayurvedic or nutritional supplements, within four weeks prior to screening. Individuals with low ASCVD risk not requiring statin therapy, those on systemic corticosteroids or hormone therapies within six weeks of screening, and pregnant or lactating women will be excluded. Smokers, individuals with poorly controlled diabetes mellitus (HbA1c > 9%), significant hepatic or renal impairment (including total bilirubin, AST, ALT, or alkaline phosphatase levels >2 × the upper normal limit, or serum creatinine >1.2 mg%), and those with a history of acute myocardial infarction or cardiac arrest within the previous 24 months are ineligible. Participants with musculoskeletal conditions limiting exercise, uncontrolled hypertension (systolic ≥160 mmHg or diastolic ≥100 mmHg), and those with co-morbidities potentially affecting study participation; such as myopathies, chronic pulmonary or endocrine diseases, systemic infections, arrhythmias, neurological or psychiatric illnesses—will be excluded. Active alcohol or substance abuse, or history thereof in the last six months, and any other medical or psychosocial condition deemed by the investigator to interfere with study integrity or participant safety, will also constitute grounds for exclusion.

### Intervention and comparator

This randomized controlled trial includes two parallel groups receiving standard statin therapy as per the 2019 ACC/AHA Guideline for the Primary Prevention of Cardiovascular Disease, with either the investigational product or a placebo administered in addition. Participants in Group I (Intervention group-TK) will receive *Trikatu* tablets (1000 mg, orally, twice daily with lukewarm water, one hour post-meal) for 12 weeks, in conjunction with standard statin therapy. Participants in Group II (comparator group) will receive a matching placebo tablets (1000 mg), identical in appearance, weight, color, and packaging to the investigational product, administered orally twice daily with lukewarm water, one hour after meals, for a duration of 12 weeks. The placebo is composed of inert microcrystalline cellulose (MCC), an excipient widely recognized for its pharmacological inertness and safety in clinical use. It has been manufactured under GMP conditions to match the physical characteristics of the *Trikatu* formulation, thereby ensuring the integrity of the double-blind study design. Participants in this group will also receive standard statin therapy as per 2019 ACC/AHA guidelines, consistent with those in the intervention arm.

#### Statin Therapy.

The statin prescribed in both groups will be Atorvastatin, administered once daily 30 minutes before dinner. The dosage—20 mg for moderate ASCVD risk and 40 mg for high ASCVD risk—will be determined based on baseline ASCVD risk assessment and clinical judgement by the consulting modern medicine physician on the research team, in accordance with the ACC/AHA 2019 guidelines and presence of comorbidities such as cardiovascular disease or diabetes mellitus.

#### Study Drug and Quality Control.

The trial intervention, *Trikatu*, has been procured from Good Manufacturing Practice (GMP) -certified Ayurvedic manufacturing pharmacies, following standard procedures for manufacturing of Churna (powder), prescribed in the Ayurvedic formulary of India (AFI). [9] All raw materials are botanically authenticated prior to processing, and quality assessment is performed in accordance with Ayurvedic pharmacopeial standards, including evaluation of relevant physicochemical parameters. Batch-to-batch consistency is ensured through standardized manufacturing processes and quality control protocols, thereby maintaining uniformity of the intervention throughout the study.

The phytochemical composition of *Trikatu* and its constituent drugs has been well documented in previous studies. Piperine, derived from Piper nigrum and Piper longum, is reported to be one of the principal bioactive constituents and is commonly used as a marker compound for quality evaluation of *Trikatu*. [10]

The dosage of *Trikatu* used in the present study was selected based on classical Ayurvedic dosage recommendations and previously published preclinical safety studies. Experimental toxicity evaluations have reported that *Trikatu* is well tolerated at therapeutic and higher dose ranges in animal models. [7,11]

Criteria for discontinuing or modifying the allocated intervention or comparator will include the occurrence of serious adverse events, clinically significant laboratory abnormalities, participant request for withdrawal, evidence of disease progression or intolerance to the study medication, or the investigator’s clinical judgment that continuation may pose undue risk to the participant’s health.

The use of any other lipid-lowering agents—including Ayurvedic or modern medicines, herbal supplements, or nutraceuticals such as omega-3 fatty acids—is prohibited during the trial to avoid confounding effects on lipid levels and gut microbiota composition. Routine medications for stable co-morbid conditions such as diabetes and hypertension, as well as short-term treatments for emergent conditions like flu or infections, may be continued if deemed necessary and approved by the study investigator, provided they are not known to significantly alter gut microbial ecology.

### Outcomes

#### Primary outcome.

The primary efficacy endpoint is the change in fasting low-density lipoprotein cholesterol (LDL-C) from baseline to 12 weeks, assessed as both percent change and absolute (mean) change, in participants receiving *Trikatu* compared with placebo, when administered as an add-on to standard statin therapy.

#### Secondary outcomes.

Secondary efficacy endpoints include the absolute change from baseline in fasting LDL-C at 12 weeks; the percent change from baseline in total cholesterol, high-density lipoprotein cholesterol, and triglyceride levels at 12 weeks; and the proportion of participants achieving fasting total cholesterol levels below 200 mg/dL at the end of the intervention period.

Secondary endpoints also include changes from baseline in gut microbiota composition at week 12, assessed using shotgun metagenomic sequencing of fecal samples. Total microbial DNA will be extracted using validated commercial kits following manufacturer-recommended protocols. Libraries will be prepared and sequenced on a next-generation sequencing platform with a target sequencing depth sufficient to ensure robust taxonomic and functional profiling.

Bioinformatic analysis will be conducted using standard, validated pipelines for quality control, host DNA removal, taxonomic profiling, and functional annotation. Microbial diversity will be evaluated using α-diversity indices (Shannon and Simpson indices) and β-diversity measures, while differential relative abundance of key bacterial taxa will be assessed. Functional metabolic pathway analysis relevant to lipid metabolism and inflammation will be predicted using curated reference databases.

Changes in gut microbiota–derived short-chain fatty acids (SCFAs), including acetate, propionate, and butyrate, will be quantified from fecal samples using gas chromatography–mass spectrometry (GC–MS) following standard derivatization procedures. SCFA concentrations will be expressed as μmol/g of fecal matter and analyzed to evaluate shifts in microbial metabolic activity associated with the intervention.

Changes in metabolic biomarkers such as adiponectin, ghrelin, apolipoprotein A1, and apolipoprotein B; and changes in inflammatory markers including high-sensitivity C-reactive protein (hs-CRP), tumor necrosis factor-α (TNF-α), and interleukin-6 (IL-6) are also included as secondary outcome measures. Role of intervention on Glycemic parameters will be evaluated through changes in fasting blood glucose, HbA1c, and fasting insulin levels, with insulin resistance estimated using the homeostatic model assessment for insulin resistance (HOMA-IR). Resting blood pressure (measured in triplicate) will be assessed at baseline, week 6, and week 12. Safety and adherence will be monitored throughout the study using structured compliance forms completed at each visit, along with systematic documentation of all participant-reported adverse events (AEs) or adverse drug reactions (ADRs).

### Harms

Harms will be defined as any untoward medical occurrence, including AEs and ADRs, whether or not they are causally related to the intervention. These will be assessed systematically at each study visit through structured interviews, physical examinations, and review of clinical laboratory parameters. Participants will be instructed to report any symptoms or health changes between visits. All reported AEs and ADRs will be recorded in case report forms and evaluated for severity, duration, seriousness, and relationship to the intervention by the study investigators. Serious adverse events (SAEs) will be reported immediately to the Institutional Ethics Committee, as per the set timelines.

### Sample size

The sample size for this study was calculated based on the expected mean change in LDL-C levels following treatment. A previous study evaluating the efficacy and safety of atorvastatin in patients with hypercholesterolemia reported an approximate 35% reduction in LDL-C levels [12]. In the present study, we hypothesized a minimum 45% reduction in LDL-C levels in the group receiving atorvastatin combined with the trial intervention, representing a clinically meaningful absolute difference of 10% between the two groups. Assuming a standard deviation of 30 mg/dL, a power of 80%, and a two-sided significance level of 5%, the required sample size was estimated to be 72 participants per group. Accounting for a 20% attrition rate, the final sample size was adjusted to 85 participants per group. Accordingly, a total of 170 participants will be enrolled in the trial.

### Participant timeline

The participant timeline includes six scheduled visits: a screening visit, baseline on Day 0 (Visit 1), and subsequent follow-ups on Day 28 (Visit 2), Day 56 (Visit 3), Day 84 (Visit 4), and Day 112 (Visit 5), with the final visit conducted without medication to evaluate sustained effects.

At the screening visit, informed consent and eligibility are assessed. Baseline assessments include demographic data, medical history, clinical examination (BP, weight, waist circumference, BMI), ECG, thyroid function (TSH), HbA1C, fasting blood sugar, inflammatory markers (Hs-CRP, TNF-α, IL-6), insulin resistance (HOMA-IR), stool sampling for gut microbiota analysis, and laboratory investigations (lipid profile, LFT, RFT, CBC). Intervention begins at baseline and continues through Day 84.

**Fig 1:**
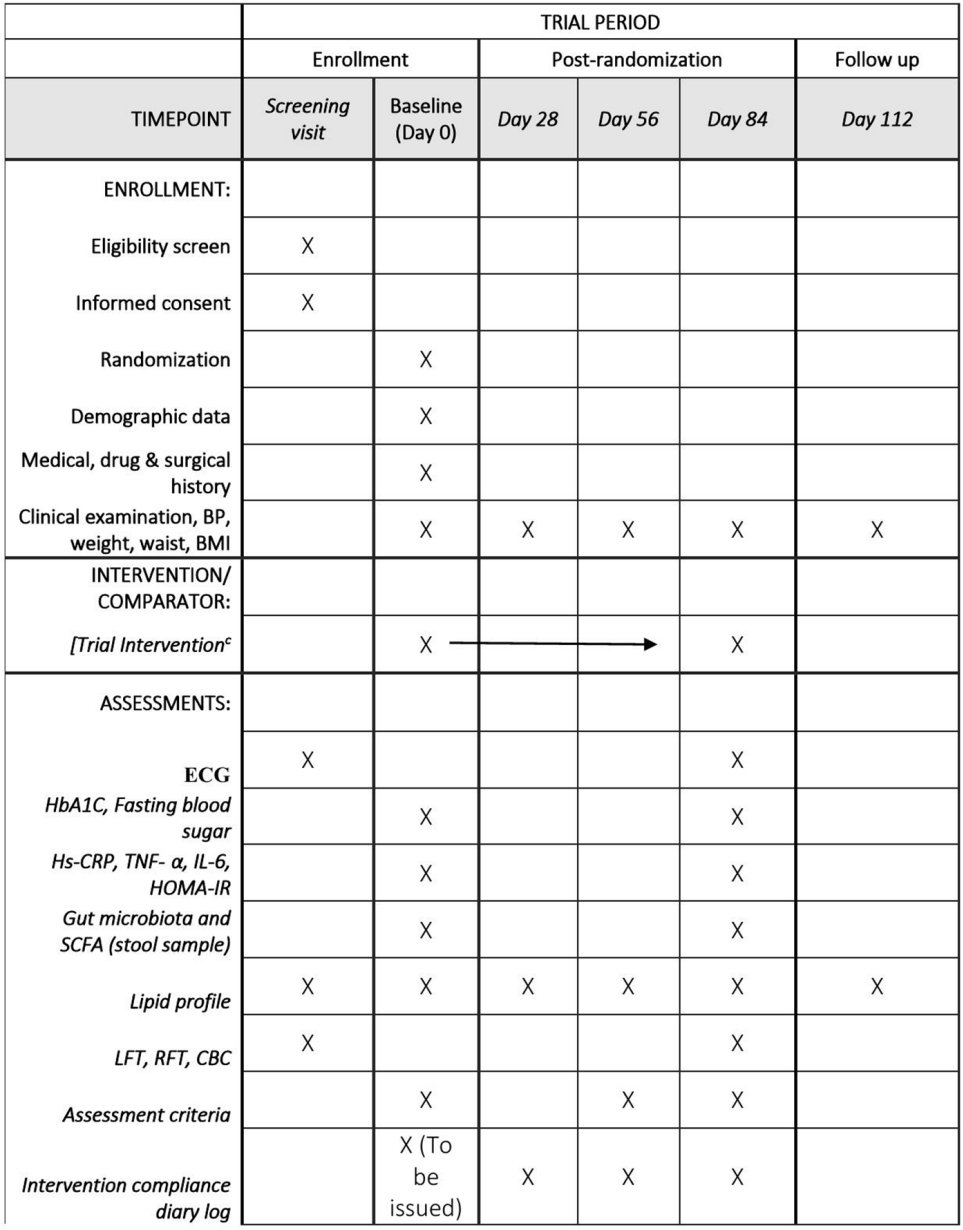
Participant timeline showing enrollment, intervention, and assessments.

Follow-up assessments involve clinical measurements, lipid profiles (Days 1, 28, 56, 84, 112), compliance tracking, and adverse event monitoring. Stool samples are recollected for gut microbiome analysis on Day 84. A repeat ECG and comprehensive labs are done on Day 84. The schedule of enrolment, interventions, and assessments for this trial is presented in accordance with the SPIRIT schedule are shown in Fig 1.

The schedule outlines screening, baseline, intervention period, and follow-up visits, with assessments conducted at specified timepoints.

### Status of study

- Participant recruitment: Currently ongoing, with an anticipated completion by April, 2026
- Data collection: ongoing, and expected to be completed by July, 2026
- Results: expected by September 2026

### Recruitment

Participants are being recruited from the outpatient departments of AIIMS Bhubaneswar. Recruitment strategies will include physician referrals, prescreening of OPD records, and display of study information through posters and leaflets. A trained research team member will facilitate screening, consent, and scheduling. Recruitment will be monitored regularly to ensure timely enrollment of the required sample size.

### Randomization

#### Sequence generation.

##### Randomization and allocation concealment:

The random allocation sequence will be generated by an independent biostatistician using a computer-generated block randomization method with variable and unequal block sizes to minimize predictability. The randomization will follow a restricted design without stratification. To ensure allocation concealment and prevent selection bias, the specific details of block sizes and sequence generation will remain confidential and are documented separately, inaccessible to the investigators and site staff responsible for participant enrollment and intervention assignment.

Implementation of the allocation sequence will be carried out centrally. Each intervention and matching placebo will be pre-packaged in identical boxes, coded according to the randomization list. The packaging and labeling of these boxes—linked only to a unique participant enrollment ID—will be performed at the central biostatistical unit of the Central Council for Research in Ayurvedic Sciences. These coded intervention kits will then be dispatched to the study site, where the investigators and treating physicians will be blinded to group assignments. The study medications will be dispensed strictly according to the participant’s sequential enrollment ID, with no access to the randomization code by site personnel, thereby preserving the double-blind design of the trial.

### Blinding

Blinding will be maintained for participants, care providers, investigators, outcome assessors, and data analysts. The trial intervention and placebo will be identical in appearance, packaging, and labeling to ensure that group allocation remains concealed throughout the study.

Unblinding will only be permissible in exceptional circumstances where knowledge of the treatment assignment is essential for the clinical management of a serious adverse event. In such cases, the decision to unblind will be made by the principal investigator in consultation with the IEC. The procedure for unblinding will involve accessing the sealed master randomization code, maintained securely by an independent third party not involved in the conduct of the trial. All instances of unblinding will be documented and reported to the ethics committee.

### Data collection, management, and analysis

#### Data collection methods.

All trial data will be systematically collected using standardized Case Report Forms (CRFs) and electronic Case Report Forms (e-CRFs) specifically designed for this study. The CRFs capture detailed information on participant demographics, medical history, intervention adherence, outcome measures, adverse events, and laboratory findings.

Study investigators, co-investigators, and designated research personnel underwent comprehensive training sessions on the study protocol, data collection procedures, and source documentation requirements prior to initiation of participant recruitment. This training was aimed at ensuring consistency in data capture, adherence to protocol-specific timelines, and compliance with Good Clinical Practice (GCP) standards. All laboratory investigations, including lipid profile, inflammatory markers, glycemic indices, and gut microbiome analysis, will be conducted in NABL-accredited laboratories using validated instruments and standardized protocols. The gut microbiota analysis will utilize metagenomic sequencing and SCFA profiling, conducted under quality-controlled conditions.

To promote participant retention and ensure complete follow-up, regular reminders via phone calls or messages will be used to maintain engagement and schedule visits. Study staff will maintain a supportive and responsive communication channel to address concerns, encourage adherence, and reinforce the importance of follow-up assessments. For participants who discontinue or deviate from the intervention protocol, efforts will be made to collect outcome data at all subsequent scheduled visits, including primary and secondary endpoints such as lipid profile, inflammatory markers, glycemic indices, and gut microbiota composition. Reasons for withdrawal or deviation will be documented in detail, and participants will be encouraged to complete end-of-study assessments, even if they do not continue the assigned treatment.

Data accuracy will be further supported by regular monitoring, cross-verification of source documents with CRFs, and automated validation checks embedded in the e-CRF platform.

#### Data management.

Data will be coded, de-identified, and stored securely with restricted access. Regular data audits will be conducted to maintain quality.

#### Statistical methods.

A comprehensive Statistical Analysis Plan (SAP) will be finalized prior to unblinding the randomization code. All analyses will be conducted following the modified intention-to-treat (mITT) principle, including all randomized participants who completed the intervention period and had evaluable data for gut microbiota, metabolic and inflammatory markers, and lipid profile assessments at the 84th day. Per-protocol (PP) analyses will also be performed as supportive. Continuous outcomes will be summarized using means, standard deviations, medians, interquartile ranges, and minimum/maximum values. Categorical variables will be presented as frequencies and percentages. No imputation will be applied for missing data; all analyses will be based on observed data.

For the primary outcome (change in fasting LDL-C from baseline to 12 weeks, expressed as percent and absolute change), between-group comparisons will be conducted using analysis of variance (ANOVA) when baseline LDL-C values are comparable between groups. In the presence of baseline variability, analysis of covariance (ANCOVA) will be employed, with treatment group as the fixed effect and baseline LDL-C included as a covariate to adjust for baseline differences.

Similarly, for secondary continuous outcomes measured at baseline and 12 weeks (total cholesterol, HDL-C, triglycerides, metabolic biomarkers, inflammatory markers, glycemic parameters), ANOVA will be applied when baseline values are balanced, while ANCOVA adjusted for baseline values will be used if baseline variability is observed.

For outcomes assessed at multiple time points, repeated-measures analysis of variance (r-ANOVA) will be used when assumptions of sphericity and normality are satisfied. If r-ANOVA assumptions are not met, linear mixed-effects models with fixed effects for treatment group, time, and group-by-time interaction, will be employed as an alternative.

Prespecified subgroup analyses will explore differential effects by baseline cardiovascular risk (e.g., Framingham Risk Score), comorbidity status, and concomitant medications. Safety assessments, including AEs and SAEs, will be summarized descriptively by treatment group, with frequencies and proportions reported. All statistical analyses will be performed using statistical software SPSS 29.0, and a significance level of 5% (two-sided) will be considered for hypothesis testing unless otherwise specified.

### Monitoring

#### Data monitoring committee.

A formal independent Data Monitoring Committee has not been established for this study, as the interventions involved are considered low risk and the study interventions are classical Ayurveda formulations in use. However, to ensure oversight and quality assurance, CCRAS will nominate a Clinical Monitoring Committee responsible for site monitoring and review of protocol adherence.

This committee, composed of designated CCRAS representatives and monitors, will conduct periodic visits to the study site to inspect facilities, verify source documents, review CRFs, and assess compliance with the protocol and GCP guidelines. The committee will work in coordination with the investigators to resolve any discrepancies or issues identified during monitoring. All data will be reviewed centrally at CCRAS to ensure consistency, completeness, and accuracy of the data. No conflicts of interest are declared by the monitoring team.

### Ethics

#### Research ethics approval.

The study protocol, case report forms (CRF), and participant information sheet (PIS) were reviewed and approved (Ref no. T/EMF/Biochem/22/15 by the Institutional Ethics Committee on 9^th^ June 2022. The study protocol is enclosed as a S1 File. The protocol was registered with the Clinical Trials Registry of India (CTRI) (registration number CTRI/2023/04/051942) Registered on 25/04/2023. The CTRI registration is enclosed as a S2 File. The trial will be conducted in accordance with the *National Ethical Guidelines for Biomedical and Health Research Involving Human Participants* (ICMR, 2017) and the *Good Clinical Practice Guidelines for Clinical Trials in Ayurveda, Siddha, and Unani Medicine (GCP-ASU), 2013*. Written informed consent will be obtained from all eligible participants prior to enrollment. In cases where a participant is temporarily unable to provide consent, consent will be sought as soon as capacity is regained. Participants and their legally authorized representatives will be informed of their right to withdraw from the study at any time and to withhold the use of their data.

### Protocol amendments

All significant protocol modifications, including changes to study design, eligibility criteria, outcomes, or study procedures will be promptly communicated to the Institutional Ethics Committee, and CTRI, CCRAS, and all relevant study personnel. The initial cut-off of HbA1c was 9% which was increased to 11% for inclusion of patients with diabetes mellitus. The IEC approval for the same was obtained on 7^th^ February 2023. Any futher approved amendments will also be updated in the protocol documents and participant materials as appropriate.

### Consent

Informed consent will be obtained by the study research fellow or designated investigator trained in the study protocol. The process will involve providing potential participants with a detailed PIS and explaining the study procedures, risks, and rights in a language they understand. Additional consent will be obtained, as outlined in the PIS, for the collection, storage, and future use of biological specimens, including fecal samples, for ancillary studies.

### Confidentiality

All personal information of potential and enrolled participants will be handled in strict confidence. Data will be collected using coded identifiers rather than names, and any documents linking participant identity to the study ID will be stored separately in a secured location with restricted access. Electronic records will be password-protected and encrypted. Only authorized study personnel will have access to identifiable data, and all study-related information will be shared in de-identified or aggregate form. Confidentiality will be maintained throughout the trial and preserved during data analysis, publication, and long-term archiving, in accordance with ethical guidelines and institutional policies.

### Ancillary and post-trial care

Participants will receive appropriate medical care for any adverse events or health issues arising from trial participation, as per institutional and regulatory guidelines. In the event of any trial-related injury or harm, compensation will be provided in accordance with applicable ethical norms and national regulations. Although no formal ancillary or post-trial care is planned beyond the study period, participants will be referred to the appropriate healthcare services as needed for continued care.

## Results

The screening and recruitment process for this trial was started on 19.07.2023. As of June 2025, 150 patients have been enrolled in both groups. It is an ongoing study and the results will be published afterward. The results of this trial will be shared with both the scientific and medical communities, irrespective of the outcome.

## Discussion

Dyslipidemia plays a central role in the pathogenesis of atherosclerotic cardiovascular disease and is a major modifiable risk factor contributing to the global burden of cardiovascular morbidity and mortality. The etiological factors for dyslipidemia include a complex interplay of poor dietary habits, sedentary lifestyle, tobacco use, and genetic predisposition [13].

Recent advances in molecular biology have highlighted the role of adipokines—bioactive peptides secreted by adipose tissue—in lipid metabolism and systemic inflammation. Molecules such as Adipolin (CTRP12) and Secreted Frizzled-Related Protein 5 (SFRP5) have emerged as key regulators of metabolic homeostasis, influencing pathways related to insulin sensitivity, glucose utilization, fatty acid oxidation, and inflammatory signaling. Dysregulation of these pathways contributes to the development and persistence of dyslipidemia and associated metabolic syndromes [14,15].

Large-scale epidemiological and interventional studies have consistently demonstrated that lowering LDL-C levels results in a substantial reduction in cardiovascular events. Meta-analyses indicate that for every 1 mmol/L reduction in LDL-C, there is approximately a 30% reduction in major adverse cardiovascular events (MACE) [16]. This effect underscores the importance of initiating lipid-lowering interventions early in the disease course—even from a younger age—in order to attenuate cumulative exposure to elevated LDL-C and associated vascular damage.

However, despite the effectiveness of pharmacologic agents such as statins, ezetimibe, and PCSK9 inhibitors, residual cardiovascular risk remains, and long-term adherence and tolerability issues persist. Healthcare systems, particularly in low- and middle-income countries, often face barriers in implementing widespread lipid management due to economic, infrastructural, and accessibility constraints. These challenges necessitate the exploration of safe, accessible, and cost-effective complementary strategies to support metabolic correction and lipid control.

This study protocol outlines the framework of a stringent, randomized, double-blind, placebo-controlled trial intended to systematically assess the efficacy and safety of *Trikatu* as an additional treatment for dyslipidemia.

This trial is expected to produce data in favour of integrating *Trikatu*, a standardized Ayurvedic Formulation, in conjunction with conventional therapy to treat dyslipidaemia. We hypothesize that the intervention may induce favorable changes in metabolic processes, leading to modulation of metabolic and inflammatory markers, which in turn may translate into a statistically and clinically meaningful reduction in LDL-cholesterol levels. Individuals who are statin intolerant, have a higher residual cardiovascular risk despite standard medication, or are searching for integrative therapeutic choices might find such data particularly valuable. Importantly, a critical assessment of *Trikatu’s* tolerability and safety will also be conducted.

In addition to its effectiveness, this study investigates the mechanistic aspects of *Trikatu* via gut microbiota analysis. Due to its potential to affect metabolic transformation, as per the specified properties in Ayurveda classics, *Trikatu* might influence the gut-lipid axis by altering microbial diversity and composition-possibly enhancing beneficial SCFA-generating genera while decreasing pro-inflammatory taxa. Showing such alterations would provide a credible biological mechanism comprehension.

## Limitations

Despite its double blind placebo controlled model, several limitations need to be acknowledged. Being a single-centre trial, the external validity of results might be restricted, and while the 12-week duration is adequate for biomarker evaluation, it may not reflect long term cardiovascular effects. Although participants will be encouraged to adhere to their regular diet and lifestyle, total control over these confounding factors is not feasible.

## Supporting information

S1 File Project Protocol: Full study protocol. (PDF)

S2 File SPIRIT Checklist: Completed checklist in accordance with SPIRIT guidelines. (DOCX)

## Abbreviations

AIIMS: all India institute of medical sciences
ASCVD: atherosclerotic cardiovascular disease
CAD: coronary artery disease
IEC: institutional ethics committee
CRF: case record form.
