# Correction: Efficacy and safety of the ayurvedic formulation ‘*Trikatu*’ as an add-on to standard care in dyslipidemia: Study protocol for a randomized, double-blind, placebo-controlled trial evaluating lipid parameters, and gut microbiota

**DOI:** 10.1371/journal.pone.0351663

**Published:** 2026-06-12

**Authors:** Shruti Khanduri, Sophia Jameela, Suchanda Sahu, Sujata Devi, Bhogavalli Chandra Sekhara Rao, Kshirod Ratha, Richa Singhal, Latika Kaushik, Narayanam Srikanth, Rabinarayan Acharya

There is an error in affiliation 7 for author Rabinarayan Acharya. The correct affiliation 7 is: Director General, Central Council for Research in Ayurvedic Sciences, Ministry of Ayush, New Delhi, India.
