# Supplementary material for: Efficacy and safety of the ayurvedic formulation ‘Trikatu’ as an add-on to standard care in dyslipidemia: Study protocol for a randomized, double-blind, placebo-controlled trial evaluating lipid parameters, and gut microbiota
Source: PLoS One. 2026 May 11;21(5):e0348058. doi: 10.1371/journal.pone.0348058 (PMC13160312; doi:10.1371/journal.pone.0348058)
Supplement: S1 File — (PDF) [file pone.0348058.s001.pdf]

PROPOSAL FOR INTRA MURAL COLLABORATIVE CLINICAL RESEARCH PROJECT FOR

Efficacy and safety of Ayurveda Formulation 'Trikatu' as add-on to standard care in Dyslipidemia – A Randomized Controlled Trial

Submitted by :

CARI, Bhubhaneswar

1. **Dr. K.K. Ratha**
Research Officer (Ayurveda), Principal Investigator

2. **M.M. Rao**
Director, Co-Investigator

AIIMS, Bhubaneswar

1. **Dr. Suchanda Sahu**
Additional Professor,
Department of Biochemistry,
Principal Investigator

2. **Dr. Sujata Devi**
Assoc. Prof.,
General Medicine, Co-Investigator

Protocol Developed and Sponsored by

CENTRAL COUNCIL FOR RESEARCH IN AYURVEDIC SCIENCES
Ministry of AYUSH, Government of India
61-65, Institutional Area, Opposite "D" Block,
Janakpuri, New Delhi - 110058

SECTION -C

SUMMARY OF THE PROPOSED PROJECT

Title: "Efficacy and safety of Ayurveda Formulation 'Trikatu' as add-on to standard care in Dyslipidemia- A Randomized Controlled Trial "

1.	Type/Category of the project (Clinical Research, Pharmacology, Chemistry, Botany, Literary, any other)	Randomized controlled trial
2.	Mandate of Institute	Clinical research focusing on Hepatobiliary disorders.
3.	Name & qualification of the Investigator (With complete Address, Ph. No./Mobile No. & E-mail etc.)	1. Dr.Kshirod Kumar Ratha, R.O. (Ay)<br>Qualification: BAMS, M.D (Ay)<br>Area of interest/ specialization: Dravyaguna<br>Central Ayurveda Research Institute for Hepatobiliary disorders., Bhubanesw<br>Odisha<br>Telephone/ Mob.: 0674 - 2387703 / 9874612213<br>E-mail: drkkratha@gmail.com, drkkratha@rediffmail.com<br>2. Dr. Suchanda Sahu<br>Additional Professor,<br>Department of Biochemistry,<br>Area of interest/ specialization: Biochemistry<br>Postal address: AIIMS, Sijua, Bhubaneswar<br>Mob:9438884031, E-mail: biochem_suchanda@aiimsbhubaneswar.edu.in
4.	Name & Qualification of the Co-Investigators. (s) (With complete Address, Ph. No./Mobile No. & E-mail etc.)	1.Name: Dr. Mrutyumjaya Rao R.O. (Ay)<br>Qualification: BAMS, M.D (Ay)<br>Area of interest/ specialization: Salyatantra<br>Postal address: Central Ayurveda Research Institute for Hepatobiliary disorders., Bhubaneswar, Odisha<br>Telephone/ Mob.: 06742386702(O), 9040224463(M)<br>**E-mail :**nriadd_bhubaneswar@gov.in, meda_mrao@yahoo.co.in<br>2. Dr. Sujata Devi<br>Assoc. Prof., General Medicine<br>Area of interest/ specialization: Internal Medicine<br>Postal address: AIIMS, Sijua, Bhubaneswar<br>Mob: 9438884203<br>E-mail: genmed_sujata@aiimsbhubaneswar.edu.in

5.	Complete Postal address of the Institute/Organization responsible for the project	Central Council for Research in Ayurvedic Sciences,Janakpuri,New Delhi
6.	Complete postal address of other Participating institutes.	1. Central Ayurveda Research Institute, Bhubaneswar.<br>At & Post – Bharatpur, Near Kalinga Studio, Bhubaneswar-751029<br>2. AIIMS, Sijua, Bhubaneswar, Odisha
7.	Project cost	**Rs.87,18,588/-** (Rupees Eighty Seven lakh eighteen thousand five hundred eighty eight only)
8.	Duration of the project	02 years
9.	**Budgetary requirement**	**Head wise requirement along with justification**

**Particulars**	**1st year**	**2nd year**	**Total**	**Justifications**
Publication in peer reviewed journals	0	300000	300000	Protocol publication in the first year and study publication after statistical analysis<br>Publication/open access charges
SRF (Ayurveda)<br>(35,000+18% HRA= @41,300/- pm)	495600	495600	991200	Assisting the PI with screening, data documentation in CRF & e-format etc
Lab Technician @16,000/pm	192000	192000	384000	For stool sample collection, blood sample collection, storage etc.
Trial drug (Atorvastatin 20mg/40mg)	200000	200000	400000	To procure trial drugs of standard care (By the AIIMS team) @ Rs.250/10 tab approx.<br>For 170 patients= 170 x 90 tab (Rs.2250/-)= Rs.382500/-<br>Rounded off to 4 lakh
**Laboratory Investigation** (ECG ,TFT, KFT, LFT, LIPID profile, FBS, HBA1C,	5287800	0	5287800	To be performed at AIIMS Biochemistry/Central

10.		Hs-CRP, CBC, Grehlin, Leptin, TNF-Alpha, Adiponectin, Apolipoprotein A1 Apolipoprotein B, IL-6, Insulin, Gut microbiota)*				lab. and one test to be outsourced (Cost X Times X No. Of Subjects)
		Reagents & solutions for preservation of stool sample, vials & tubes etc. for sample collection.	1000 00	1000 00	20000 0	Reagents & solutions for preservation of stool sample (eg: modified Cary Blair medium), Vials & syringes, EDTA tubes for Blood collection, aliquots, stool collection pan, sample storage equipment, sample transport accessories are required
		**Stationary**	1500 00	1000 00	25000 0	For purchase of files, paper, register, computer cartridge, printing of CRF
		**Travelling support/incidental support expenses for research subject**	8500 0	0	85000	To be paid to research subject on each visits for 5 visits. (100 x 5 visits x 170 subjects)
		**Local TA/conveyance for investigator/Honoraria for IEC member including conveyance**	5000 0	5000 0	10000 0	For local TA of project investigators, personnel to camp sites, TA/conveyance for investigator/Honoraria for IEC member including conveyance
		**Contingency or Miscellaneous expenses**	1000 00	5000 0	15000 0	To meet unforeseen expenses
		**Total**	6660 400	1487 600	81480 00	
		**Overhead Institutional charges 5%**	3330 20	7438 0	40740 0	
		**Grand Total**	6993 420	1561 980	85554 00	

**Grand Total**	**Rs.85,55,400/- (Rupees Eighty five lakh fifty five thousand four hundred only)**

Technical part

a. Background

Lipid disorders involving derangements in serum cholesterol, triglycerides, or both are commonly encountered in clinical practice and often have implications for cardiovascular risk and overall health. Dyslipidaemias are collectively among the most commonly detected lifestyle or chronic metabolic disorder. They are classically characterized by abnormal serum levels of cholesterol, triglycerides, or both, involving abnormal levels of related lipoprotein speciesi. The most common risk factor and cause for mortality and morbidity associated with dyslipidaemia is associated atherosclerotic cardiovascular disease (ASCVD). Dyslipidemias are an active and expanding area of research, with recent studies providing insight into their molecular basis and genetic origins, outlining their role in the development of atherosclerosis, and clarifying the ability of pharmacologic agents to ameliorate ASCVD risk in affected individuals. There is a strong pathophysiological association of raised LDL cholesterol with initiation and progression of coronary atherosclerosis and contemporary clinical evidence shows that lowering its levels can regress and stabilize atherosclerotic vascular disease. Treatment of dyslipidaemia is the most effective modifiable target for improving cardiovascular outcomes.

In India, only limited studies exist on epidemiology of cholesterol and other lipoprotein lipids on large samples in the last 20 years[ii, iii].Studies from India have reported greater triglyceride levels in rural and urban populations associated with low HDL cholesterol levels[iv]. The low HDL cholesterol and hypertriglyceridemia are metabolically interlinked and their combination has been termed as atherogenic dyslipidemia, which is also associated with increased levels of small-dense LDL particles and insulin resistance[v].

The prevalence rates of various fasting dyslipidemia in the first phase of ICMR INDIAB study restricted to urban and rural populations in 4 states in India was hypercholesterolemia in 13.9%, high triglycerides in29.5%, low HDL cholesterol in 72.3% and high LDL cholesterol in11.8%. 79% men and women had abnormalities in at least one of the lipid parameters[vi]. Focus

on dyslipidemia management is urgently required in India to halt the rising tide of coronary heart disease.

When lifestyle interventions alone are not enough to correct dyslipidemia, or those who are determined to be at sufficient ASCVD risk, lipid modifying drugs are used after a clinician-patient overall discussion. In most patients, currently available lipid modifying drugs are effective in bringing the lipid levels to goal thereby reducing the risk of CV events[vii]. The general approach to management is that in young adult patients (20 - 39 years) promoting a healthy lifestyle can suffice and improve dyslipidemia. Pharmacological therapy in this population is usually indicated in patients with LDL-C levels (starting from 160 mg/dL or higher than 100 mg/dL in high risk patients).

Dietary interventions appear to be a promising strategy for managing premature hyperlipidemia. In a trial, a probiotic formulation resulted in decreased LDL-C and improvements in triglyceridemia and HDL cholesterol (HDL-C) levels. Mechanisms that support the potential efficacy of the abovementioned interventions include the suppression of liver cholesterol synthesis, reduction of intestinal cholesterol absorption and production of conjugated linoleic acid in the gut[viii]. Expanding scientific evidence indicates that the gut microbiota mediates pathophysiological mechanisms that alter lipid metabolism and other related metabolic traits[ix]. Particularly, the intestinal microbiota has been recognized as a metabolically active endocrine organ of the human superorganism that can be a therapeutic target for hyperlipidemia and associated cardio-metabolic diseases[x].

"Trikatu"-an Ayurvedic formulation comprising of a 1:1:1 ratio of dried fruits of *Piper nigrum, Piper longum* and dried rhizomes of *Zingiber officinale* is widely used in Ayurveda clinical practice. In Ayurvedic Formulary of India it is used in various diseases like, Arocaka (Tastelessness), Agnimandya (Digestive impairment), Amadosa (Products of impaired digestion and metabolism / consequences of Ama), Gala Roga (Diseases of throat), Pinasa (Chronic rhinitis/sinusitis), Kustha (Diseases of skin), Swasa (Dyspnoea/Asthma), Kasa (Cough), Tvakroga (Skin disease), Gulma (Abdominal lump), Meha (Excessive flow of urine), Sthaulya (Obesity), Slipada (Filariasis)[xi]. Clinical evidence from a study on Trikatu in

dyslipidemia depicts statistically significant improvement in the parameters of lipid profile and obesity when administered for a period of 8 weeks[xii].

This study is planned with the objective to utilize the anti-dyslipidemic activity of Trikatu to effectively improve the lipid metabolism and to regulate the gut dysbiosis which is now considered a marker of impaired metabolism

b. Study objectives

Primary objective: To assess the efficacy and safety of Ayurvedic Formulation "Trikatu" for improving lipid parameters in dyslipidemia

Secondary Objective: To assess the Changes in Gut Microbiota in response to the trial interventions and delineate correlates that predict favorable outcome in dyslipidemia patients

c. Study setting: Participants would be screened from the Department of Medicine, AIIMS, Bhubaneswar and Ayurveda OPD of Central Ayurveda Research Institute, Bhubaneswar. All investigations of the study subjects will be carried out at Biochemistry Department and Central laboratory, AIIMS, Bhubaneswar. All study procedures following enrollment will be done at AIIMS, Bhubaneswar. The gut microbiota analysis will be outsourced.

d. Study Population: Patients with Dyslipidaemia diagnosed based on the 2019 ACC/AHA Guideline on the Primary Prevention of Cardiovascular Disease and required for statin therapy will only be selected.

e. Inclusion criteria:

- Participants of any gender in the age group 25-60 years
- Diagnosis of moderate and high ASCVD risk dyslipidemia based on the ACC/AHA Guidelines, 2019 on the Primary Prevention of Cardiovascular Disease and indicated for statin therapy
- Body Mass Index (BMI) [illegible] 18.5 and < 34.9 kg/m².
- Written informed consent provided prior to screening, after receiving and understanding the subject information.
- Willingness to adhere to the treatment for a period of 12 weeks

Exclusion criteria:

- Subjects on any lipid altering drug therapy other than statins (including any contemporary, Ayurvedic or traditional medicines and nutritional supplements such as Omega-3 Fatty acids, anti-obesity drugs) within 4

weeks prior to screening*.

- Patients of dyslipidaemia with low ASCVD risk as per the ACC/AHA guidelines, 2019 and not indicated for statin therapy
- Subject using or were using the following medications: systemic corticosteroids, cyclical or non-continuous hormone therapy (estrogen or testosterone). within 6 weeks prior to screening
- Women who are pregnant or breastfeeding, or planning a pregnancy during the duration of the study.
- Smoker
- Diabetes mellitus, uncontrolled despite the use of anti-diabetic medication (HbA1c>9)
- Clinically significant liver and/or an elevation in either total bilirubin, alkaline phosphatase, AST, ALT of >2 times the laboratory reference
- Chronic kidney diseases having serum creatinine >1.2 mg%
- Diagnosis of acute myocardial infarction or cardiac arrest within previous 24 months.
- Musculoskeletal disease involving the lower limbs (severe painful arthritis, fracture, or joint contracture) which would impede the participant from adhering to the exercise regime.
- Uncontrolled hypertension defined as evidenced by a reproducible (repeated 5 minutes apart) sitting BP ≥160 systolic or ≥100 mmHg diastolic
- Co-morbid conditions that would interfere with study activities or response to treatment, which may include: Myopathies, Severe chronic pulmonary disease, thyroid disorders, endocrine diseases, Local, systemic acute or chronic infectious illness, cardiac arrhythmias, Neurological, psychological illness.
- Active substance abuse like alcohol or drug abuse in any form or history of alcohol/drug abuse or dependence within 6 months prior to study enrolment, or inability to refrain from alcohol use
- History or evidence of any other clinically significant disorder, condition or disease that, in the opinion of the investigator would interfere with the study evaluation, procedures, or completion.
- Any other condition, that as per the PI would jeopardize the study/ study outcomes

f. Trial treatment:

Group I : Tablet Trikatu 1000 mg twice daily with luke warm water one hr after food for 12 weeks along with standard care (Statins: dose/ intensity of Statins as per 2019 ACC/AHA Guideline)

Group II: Matching placebo 1000 mg twice daily with luke warm water one hr after food for 12 weeks along with standard care (Statins: dose/ intensity of Statins will be adjudicated as per 2019 ACC/AHA Guideline)

g. Outcome Measures:

Primary Outcome Measure:

- Change in fasting serum LDL cholesterol [Time Frame: 0, 12 weeks]

Secondary Outcome measures:

- Change in fasting Total cholesterol [Time Frame: 0, 12 weeks]
- Changes in the Gut microbiota profile (Time frame: 0, 12 weeks)
- Proportion of participants in the normal reference range (less than 200 mg/dl) for fasting T. cholesterol [Time Frame: 12 weeks]
- Change in fasting HDL-cholesterol [Time Frame: 0, 12 weeks]
- Change in fasting Triglycerides [Time Frame: 0, 12 weeks]
- Change in Adiponectin, ghrelin, apolipoprotein A1 and apolipoprotein B levels
- Change in Hs-CRP, tumor necrosis factor-α (TNF-α) and interleukin-6 (IL-6)
- Change in Resting blood pressure (measured in triplicate) [Time Frame: 0, 6, 12 weeks]
- Change in fasting glucose & HbA1C [Time Frame: 0, 12 weeks]
- Change in fasting insulin [Time Frame: 0, 12 weeks)
- Improvement in homeostatic model assessment to quantify insulin resistance (HOMA-IR) [Time Frame: 0, 12 weeks]
- Drug compliance (elicited at each visit, in a structured compliance reporting form)
- Reported AE/ADR (Participant reported AE/ADR recorded in structured formats)

h. Timeline:

Estimated Study Duration: 2 years

- **Preparatory period:** 08 weeks

		➢ Recruitment of project personnel,<br>➢ IEC approval<br>➢ Preparation of CRF & e-Format<br>➢ Training of personnel<br>➢ Procurement of Medicines<br>• **Study intervention (for participants)** : 12 weeks (84 days)<br>• **Follow-up:** 04 weeks<br>• **Recruitment period:** 60 weeks<br>• **Statistical analysis & Publication:** 12 weeks

Section - D

Detailed Research Protocol

Efficacy and safety of Ayurveda Formulation 'Trikatu' as add-on to standard care in Dyslipidemia- A Prospective Randomized Double Blind Placebo Controlled Trial

Protocol Number: DL-22-001

Protocol version V.1 09 February 2022

Clinical Trial Protocol

Sponsored by:

CENTRAL COUNCIL FOR RESEARCH IN AYURVEDIC SCIENCES
Ministry of AYUSH, Government of India
61-65, Institutional Area, Opposite "D" Block,
Janakpuri, New Delhi - 110058

RESTRICTED CIRCULATION

Efficacy and safety of Ayurveda Formulation 'Trikatu' as add-on to standard care in Dyslipidemia- A Prospective Randomized Double Blind Placebo Controlled Trial

SPONSOR

Central Council for Research in Ayurvedic Sciences (C.C.R.A.S.),
Jawahar Lal Nehru Bhartiya Chikitsa Evam Homoeopathy Anusandhan Bhawan
61-65, Institutional Area,
Opposite D-Block, Janakpuri,
New Delhi-110058

Particulars of the participating centers & the Investigator(s)

S. No .	Site	Name and contact details of the Principal Investigator	Name and contact details of the Co-Investigator
	CARI Bhubhaneswar	Dr.K.K.Ratha,<br>Research Officer (Ayurveda)<br>Mob:9874612213<br>E-mail:drkkratha@gmail.com	Dr.M.M.Rao<br>Director<br>Mob:9348187667<br>E-mail:medamrao69@gmail.com
	AIIMS, Bhubaneswar	Dr.Suchanda Sahu<br>Additional Professor, Department of Biochemistry,<br>Mob:9438884031<br>E-mail:<br>biochem_suchanda@aiimsbhubaneswar.edu.in	Dr. Sujata Devi<br>Assoc. Prof., General Medicine<br>Mob:9438884203<br>E-mail:<br>genmed_sujata@aiimsbhubaneswar.edu.in

Protocol developed by:

Central Council for Research in Ayurvedic Sciences (C.C.R.A.S.)
Opposite D-Block, Janakpuri,
New Delhi-110058

INVESTIGATOR AGREEMENT

By signing below, I agree that:

I have read this protocol. I approve this document and I agree that it contains all necessary details for carrying out the study as described. I will conduct this study in accordance with the design and specific provision of this protocol and will make a reasonable effort to complete the study within the time designated. I will provide copies of this protocol and access to all information furnished by CCRAS to study personnel under my supervision. I will discuss this material with them to ensure they are fully informed about the study product and study procedures. I will let them know that this information is confidential and proprietary to CCRAS and that it may not be further disclosed to third parties. I understand that the study may be terminated or enrolment suspended at any time CCRAS, with or without cause, or by me if it becomes necessary to protect the best interests of the study subjects.

I agree to conduct this study in full accordance with, Institutional Ethics Committee Regulations, and ICH Guidelines for Good Clinical Practices.

______________________________ ______________

Investigator's Signature Date

____

Investigator's Name

Protocol summary

Title: Efficacy and safety of Ayurveda Formulation 'Trikatu' as add-on to standard care in Dyslipidemia- A Prospective Randomized Double Blind Placebo Controlled Trial

Principal Investigator:

Study setting: CARI, Bhubaneswar

Study objectives

Primary objective: To assess the efficacy and safety of Ayurvedic Formulation "Trikatu" for improving lipid parameters in dyslipidaemia

Secondary Objective: To assess the Changes in Gut Microbiota in response to the trial interventions and delineate correlates that predict favorable outcome in dyslipidemia patients

Study duration: 2 years

Study design: Prospective double blind randomized placebo controlled trial.

Intervention:

Group I : Tablet Trikatu 1000 mg twice daily with luke warm water one hr after food for 12 weeks along with standard care (Statins)

Group II: Matching placebo 1000 mg twice daily with luke warm water one hr after food for 12 weeks along with standard care (Statins)

The participants in both groups will receive standard care as per the 2019 ACC/AHA

Duration of intervention: 12 weeks (84 days)

Sample size: 85 per group (Total 170)

Study Population: Patients with Dyslipidemia diagnosed based on the 2019 ACC/AHA Guideline on the Primary Prevention of Cardiovascular Disease.

Background:

Lipid disorders involving derangements in serum cholesterol, triglycerides, or both are commonly encountered in clinical practice and often have implications for cardiovascular risk and overall health. Dyslipidaemias are collectively among the most commonly detected lifestyle or chronic metabolic disorder. They are classically characterized by abnormal serum levels of cholesterol, triglycerides, or both, involving abnormal levels of related lipoprotein speciesxiii. The most common risk factor and cause for mortality and morbidity associated with dyslipidaemia is associated atherosclerotic cardiovascular disease (ASCVD). Dyslipidemias are an active and expanding area of research, with recent studies providing insight into their molecular basis and genetic origins, outlining their role in the development of atherosclerosis, and clarifying the ability of pharmacologic agents to ameliorate ASCVD risk in affected individuals. There is a strong pathophysiological association of raised LDL cholesterol with initiation and progression of coronary atherosclerosis and contemporary clinical evidence shows that lowering its levels can regress and stabilize atherosclerotic vascular disease. Treatment of dyslipidaemia is the most effective modifiable target for improving cardiovascular outcomes.

In India, only limited studies exist on epidemiology of cholesterol and other lipoprotein lipids on large samples in the last 20 years[xiv, xv].Studies from India have reported greater triglyceride levels in rural and urban populations associated with low HDL cholesterol levels[xvi]. The low HDL cholesterol and hypertriglyceridemia are metabolically interlinked and their combination has been termed as atherogenic dyslipidemia, which is also associated with increased levels of small-dense LDL particles and insulin resistance[xvii].

The prevalence rates of various fasting dyslipidemia in the first phase of ICMR INDIAB study restricted to urban and rural populations in 4 states in India was hypercholesterolemia in 13.9%, high triglycerides in29.5%, low HDL cholesterol in 72.3% and high LDL cholesterol in11.8%. 79% men and women had abnormalities in at least one of the lipid parameters[xviii]. Focus on dyslipidemia management is urgently required in India to halt the rising tide of coronary heart disease.

When lifestyle interventions alone are not enough to correct dyslipidemia, or those who are determined to be at sufficient ASCVD risk, lipid modifying drugs are used after a clinician-patient overall discussion. In most patients, currently available lipid modifying drugs are effective in bringing the lipid levels to goal thereby reducing the risk of CV events[1]. The general approach to

---

https://www.journaljammr.com/index.php/JAMMR/article/view/30469/57160

management is that in young adult patients (20 - 39 years) promoting a healthy lifestyle can suffice and improve dyslipidemia. Pharmacological therapy in this population is usually indicated in patients with LDL-C levels (starting from 160 mg/dL or higher than 100 mg/dL in high risk patients).

Dietary interventions appear to be a promising strategy for managing premature hyperlipidemia. In a trial, a probiotic formulation resulted in decreased LDL-C and improvements in triglyceridemia and HDL cholesterol (HDL-C) levels. Mechanisms that support the potential efficacy of the abovementioned interventions include the suppression of liver cholesterol synthesis, reduction of intestinal cholesterol absorption and production of conjugated linoleic acid in the gut[xix]. Expanding scientific evidence indicates that the gut microbiota mediates pathophysiological mechanisms that alter lipid metabolism and other related metabolic traits[xx]. Particularly, the intestinal microbiota has been recognized as a metabolically active endocrine organ of the human superorganism that can be a therapeutic target for hyperlipidemia and associated cardio-metabolic diseases[xxi].

"Trikatu"-an Ayurvedic formulation comprising of a 1:1:1 ratio of dried fruits of *Piper nigrum, Piper longum* and dried rhizomes of *Zingiber officinale* is widely used in Ayurveda clinical practice. In Ayurvedic Formulary of India it is used in various diseases like, Arocaka (Tastelessness), Agnimandya (Digestive impairment), Amadosa (Products of impaired digestion and metabolism / consequences of Ama), Gala Roga (Diseases of throat), Pinasa (Chronic rhinitis/sinusitis), Kustha (Diseases of skin), Swasa (Dyspnoea/Asthma), Kasa (Cough), Tvakroga (Skin disease), Gulma (Abdominal lump), Meha (Excessive flow of urine), Sthaulya (Obesity), Slipada (Filariasis)[xxii]. Clinical evidence from a study on Trikatu in dyslipidemia depicts statistically significant improvement in the parameters of lipid profile and obesity when administered for a period of 8 weeks[xxiii].

This study is planned with the objective to utilize the anti-dyslipidemic activity of Trikatu to effectively improve the lipid metabolism and to regulate the gut dysbiosis which is now considered a marker of impaired metabolism

Trial Design :. A randomized, double-blind, placebo-controlled clinical trial with a treatment period of 12 weeks.

Summary of trial design:

Eligible patients will be randomized into either the TK group or the placebo group. After screening, a total of 5 visits will be scheduled at baseline (visit 1) and Day 28(visit 2), Day 56 (visit 3), and

Day 84 (visit 4) to assess treatment efficacy and safety and a final without medication follow-up on Day 112 (Visit 5) to assess the sustenance of the response obtained (if any) in the trial participants.

Study objectives

Primary objective: To assess the efficacy and safety of Ayurvedic Formulation "Trikatu" for improving lipid parameters in dyslipidemia

Secondary Objective: To assess the Changes in Gut Microbiota in response to the trial interventions and delineate correlates that predict favorable outcome in dyslipidemia patients

Study setting: Participants would be screened from the Department of Medicine, AIIMS, Bhubaneswar and Ayurveda OPD of Central Ayurveda Research Institute, Bhubaneswar. All investigations of the study subjects will be carried out at Biochemistry Department and Central laboratory, AIIMS, Bhubaneswar. All study procedures following enrollment will be done at AIIMS, Bhubaneswar. The gut microbiota analysis will be outsourced.

Study Population: Patients with Dyslipidaemia diagnosed based on the 2019 ACC/AHA Guideline on the Primary Prevention of Cardiovascular Disease and required for statin therapy will only be selected.

Estimated Study Duration: 2 years

- **Preparatory period:** 08 weeks
  - Recruitment of project personnel,
  - IEC approval
  - Preparation of CRF & e-Format
  - Training of personnel
  - Procurement of Medicines
- **Study intervention (for participants)** : 12 weeks (84 days)
- **Follow-up:** 04 weeks
- **Recruitment period:** 60 weeks
- **Statistical analysis & Publication:** 12 weeks

Inclusion criteria:

- Participants of any gender in the age group 25-60 years
- Diagnosis of moderate and high ASCVD risk dyslipidemia based on the ACC/AHA Guidelines, 2019 on the Primary Prevention of Cardiovascular Disease and indicated for statin therapy
- Body Mass Index (BMI) □ 18.5 and < 34.9 kg/m^2.

- Written informed consent provided prior to screening, after receiving and understanding the subject information.
- Willingness to adhere to the treatment for a period of 12 weeks

Exclusion criteria:

- Subjects on any lipid altering drug therapy other than statins (including any contemporary, Ayurvedic or traditional medicines and nutritional supplements such as Omega-3 Fatty acids, anti-obesity drugs) within 4 weeks prior to screening*.
- Patients of dyslipidaemia with low ASCVD risk as per the ACC/AHA guidelines, 2019 and not indicated for statin therapy
- Subject using or were using the following medications: systemic corticosteroids, cyclical or non-continuous hormone therapy (estrogen or testosterone). within 6 weeks prior to screening
- Women who are pregnant or breastfeeding, or planning a pregnancy during the duration of the study.
- Smoker
- Diabetes mellitus, uncontrolled despite the use of anti-diabetic medication (HbA1c>9)
- Clinically significant liver and/or an elevation in either total bilirubin, alkaline phosphatase, AST, ALT of >2 times the laboratory reference
- Chronic kidney diseases having serum creatinine >1.2 mg%
- Diagnosis of acute myocardial infarction or cardiac arrest within previous 24 months.
- Musculoskeletal disease involving the lower limbs (severe painful arthritis, fracture, or joint contracture) which would impede the participant from adhering to the exercise regime.
- Uncontrolled hypertension defined as evidenced by a reproducible (repeated 5 minutes apart) sitting BP ≥160 systolic or ≥100 mmHg diastolic
- Co-morbid conditions that would interfere with study activities or response to treatment, which may include: Myopathies, Severe chronic pulmonary disease, thyroid disorders, endocrine diseases, Local, systemic acute or chronic infectious illness, cardiac arrhythmias, Neurological, psychological illness.
- Active substance abuse like alcohol or drug abuse in any form or history of alcohol/drug abuse or dependence within 6 months prior to study enrolment, or inability to refrain from alcohol use
- History or evidence of any other clinically significant disorder, condition or disease that, in the opinion of the investigator would interfere with the study evaluation, procedures, or completion.
- Any other condition, that as per the PI would jeopardize the study/ study outcomes

Participant Enrolment:

Before the participant begin participation in any study-specific activities/procedures, a copy of the ethics committee (IEC) approval of the protocol, informed consent, and all other participant

information and/or recruitment material, should be ready. All participants must personally sign and date the informed consent form before commencement of study-specific activities/procedures

A participant is considered enrolled when the investigator decides that the participant has met all eligibility criteria. The investigator is to document this decision and date, in the medical record and in/on the enrollment case report form (CRF).

Each participant who participates in the screening for the study receives an identification number before any study-related activities/procedures are performed. They shall also be assigned a unique enrolment number as soon as they are enrolled in the study. This number will be used to identify the participant throughout the clinical study and must be used on all study documentation related to that participant.

Participant information and consent

Written, informed consent will be sought from all eligible participants.

If a participant was unable to give consent at trial entry, but regains capacity to receive information and give consent, he/she will be given information as soon as possible and asked whether he/she is willing to continue his/her participation in the trial. The participant and his/her legal representative will be informed of the right to withdraw from the trial and object to the use of his/her data.(Annex: Participant information sheet and Consent Form)

Participant Screening:

Screened participants could broadly be defined as participants with dyslipidemia, who present at the site during the recruitment time interval. The willing volunteers will undergo a 1-2 hour screening with assessment of medical history, laboratory examinations, physical and ASCVD risk evaluation. The screening visit may be done over the course of more than one day, if needed, for participant convenience or due to delay in obtaining test results but it is preferable to finish it within one day.

Randomization procedure

Block randomization list with unequal block sizes will be generated to randomize the participants to either receive intervention or a matching placebo in the ratio of 1:1. The packaging of the trial intervention and placebo will be done as per the randomization list and will maintain concealment and double blinded treatment allocation. After randomization neither the participant nor the investigator will be aware of the treatment allocation. Only the statistician who will generate the randomization sequence will be aware of the treatment allocation, and will keep the sequence sealed till the completion of the study.

Trial treatment:

Group I : Tablet Trikatu 1000 mg twice daily with luke warm water one hr after food for 12 weeks along with standard care (Statins: dose/ intensity of Statins as per 2019 ACC/AHA Guideline)

Group II: Matching placebo 1000 mg twice daily with luke warm water one hr after food for 12 weeks along with standard care (Statins: dose/ intensity of Statins will be adjudicated as per 2019 ACC/AHA Guideline)

The participants (moderate and high-risk group) in both groups will receive standard therapy (Atorvastatin) as required and decided by the modern consultant who is part of the research team. The need/dosage/intensity for statins would be assessed through a baseline ASCVD risk assessment /2019 ACC/AHA Guideline on the Primary Prevention of Cardiovascular Disease and associated co-morbidities such as CVD, DM etc. A dosage of 20 mg and 40 mg Atorvastatin will be prescribed respectively to moderate and high risk study participants 30 minutes before dinner.

Concomitant Therapy:

The participant shall continue any concomitant therapy for DM or HTN or any other disease during the trial, which is not specifically excluded in the protocol. The investigators may prescribe any concomitant medications or treatments deemed necessary to provide adequate supportive care during the intervention period.

The therapy name, indication, dose, unit, frequency, start date, and stop date (if applicable) for all interventions (medicine/procedure/therapy) are to be recorded on each participant's CRF or Diary.

Outcome Measures:

Primary Outcome Measure:

- Change in fasting serum LDL cholesterol [Time Frame: 0, 12 weeks]

Secondary Outcome measures:

- Change in fasting Total cholesterol [Time Frame: 0, 12 weeks]
- Changes in the Gut microbiota profile (Time frame: 0, 12 weeks)
- Proportion of participants in the normal reference range (less than 200 mg/dl) for fasting T. cholesterol [Time Frame: 12 weeks]
- Change in fasting HDL-cholesterol [Time Frame: 0, 12 weeks]
- Change in fasting Triglycerides [Time Frame: 0, 12 weeks]
- Change in Adiponectin, ghrelin, apolipoprotein A1 and apolipoprotein B levels
- Change in Hs-CRP, tumor necrosis factor-α (TNF-α) and interleukin-6 (IL-6)
- Change in Resting blood pressure (measured in triplicate) [Time Frame: 0, 6, 12 weeks]
- Change in fasting glucose & HbA1C [Time Frame: 0, 12 weeks]
- Change in fasting insulin [Time Frame: 0, 12 weeks)
- Improvement in homeostatic model assessment to quantify insulin resistance (HOMA-IR) [Time Frame: 0, 12 weeks]

- Drug compliance (elicited at each visit, in a structured compliance reporting form)
- Reported AE/ADR (Participant reported AE/ADR recorded in structured formats)

End of Study

The date on which the last participant completes the study post randomization and initiation of treatment as per the allocation into either interventional arm or control arm on Day 1.

Final completion: The end of study, is defined as the time when the last participant is assessed or participates in study procedures for evaluation in the study

STUDY PROCEDURES:

Screening assessments and study procedures outlined in this section and in Table 1 can only be performed after obtaining a signed informed consent. This includes any discontinuation of the participant's medication for the purpose of participation in this study.

It is very important to attempt to perform study procedures and obtain samples at the precise time points stipulated below. When it is not possible to perform the study visit at the exact time point, the visit may be performed within the acceptable visit window as defined in the visit-specific section below.

All study procedures for a visit should be completed on the same day. Any missed visits, tests not done, or examinations that are not conducted must be reported as such on the CRFs. Subsequent study visits should resume on the original schedule. Missed assessments at prior visits should not be duplicated at subsequent visits.

Schedule of Assessments:

Procedure	Screening visit	Baseline (Visit-1) (Day 1)	Day 28 (Visit-II)	Day 56 (Visit III)	Day 84 Visit IV)	Day 112 (Visit V) Without medicine
Informed Consent	✓					
Assessment of eligibility	✓					
Randomization	✓					
Demographic data		✓				
History taking		✓				
Clinical examination, BP, Weight, waist		✓	✓	✓	✓	✓

**circumference, BMI,**						
**Trial intervention**		✓	✓	✓	✓	
**ECG**	✓					✓
**TSH**						
**HbA1C, Fasting Blood sugar**	✓				✓	
**Hs-CRP,TNF-α & IL-6, HOMA-IR**		✓			✓	
**Gut microbiota**		✓			✓	
**Lipid Profile**	✓		✓	✓	✓	✓
**LFT, RFT, CBC**	✓				✓	
**Assessment criteria**		✓		✓	✓	
**Intervention compliance form (Diary log)**		✓(issued to participant)	✓	✓	✓	

All visits, except baseline, may have a window period of ±5 days, if the participant has any difficulty in visiting the

Clinical visits:

Baseline: Day 1

After the screening, the participants would be randomized and allocated into either of the two groups and the participant demographic details, drug history, medical history, surgical history and clinical assessment will be recorded in the CRF. The participant would be initiated into study and the intervention would be initiated as per the protocol. The general physician would prescribe the standard care as per the current guidelines after FRS assessment. The participant would be instructed to fill the therapy adherence regime in a diary log for every day till 84 days. Stool sample for gut microbiome assessment would be collected and sent for analysis.

The following procedures will be performed:

- Record concomitant medications, including statin (standard care) , dose plus adherence for prior month
- Obtain body weight
- Record vital signs (blood pressure, heart rate, respiratory rate, and temperature);
- Collect fasting blood samples for Lipid profile, Blood sugar, Adiponectin, ghrelin, apolipoprotein A1 and apolipoprotein B , Hs-CRP, IL-6,LFT, RFT etc
- Collect stool sample for gut microbiome metagenomics analysis
- FRS

- Provide trial drug for 28 days

Day 28, Day 56:

The following procedures will be performed:

- Record concomitant medications, including statin (standard care) , dose plus adherence for prior month;
- Obtain body weight, BMI, Waist circumference
- Record vital signs (blood pressure, heart rate, respiratory rate, and temperature)
- Collect fasting blood samples for Lipid profile
- Provide trial drug for 28 days;
- Assess adverse events and compliance to the trial intervention for the past 28 days

Day 84:

The following procedures will be performed:

- Record concomitant medications, including statin (standard care) , dose plus adherence for prior month
- Obtain body weight
- Record vital signs (blood pressure, heart rate, respiratory rate, and temperature);
- Collect fasting blood samples for CBC, Lipid profile, Blood sugar, Adiponectin, ghrelin, apolipoprotein A1 and apolipoprotein B , Hs-CRP, IL-6,LFT, RFTetc
- Collect stool sample for gut microbiome metagenomics analysis
- FRS
- Provide trial drug for 28 days
- Assess adverse events and compliance to the trial intervention for the past 30 days

Day 112:

The following procedures will be performed:

- Record concomitant medications, including statin (standard care) , dose plus adherence for prior month
- Obtain body weight
- Record vital signs (blood pressure, heart rate, respiratory rate, and temperature);
- Collect fasting blood samples for Lipid profile

Laboratory Investigations:

All screening and on-study laboratory samples will be processed and sent to Biochemistry Department ,AIIMS, Bhubaneswar The results of this testing will be maintained in the source documents at the site. The date and time of sample collection will be recorded in the source documents at the site. .At screening, laboratory investigations such as hematology (WBC, Hb, RBC, Hematocrit, Platelets, Differential Leucocyte Count), HbA1C, lipid profile, LFT, RFT, Thyroid profile would be done to rule out any systemic pathology, following which the participant would undergo an ECG. The results will be carried over to baseline and it is expected that the gap between screening and baseline visit is less than 2 days. Adiponectin, ghrelin, apolipoprotein A1 and apolipoprotein B, Hs-CRP, IL-6 would be assessed on baseline. Stool sample would be collected for

gut microbiome analysis at baseline and day 90. The laboratory investigations would again be done on day 90.

Sample Size and statistical considerations:

Sample size: 85 per group and a total of 170 participants

The sample size for the study has been calculated considering the mean change in LDL-c levels after treatment.. A previous study on the efficacy and safety of atorvastatin in patients with hypercholesterolemia denoted a change of 35% after treatment in LDL-c levels. We hypothesized a change of at least 45% in the LDL-c levels after treatment in patients treated with atorvastatin + trial intervention as add-on. Therefore, a mean change of 14 mg/dl (equivalent to absolute difference of 10% change between both the groups) in LDL-c levels after treatment between both groups will be considered as clinically relevant, with a standard deviation of 30 mg/dl based on the results of the above quoted study, with 80% power and 5% level of significance, a sample size of 72 participants per group was calculated. Adding an attrition rate of 20%, the final sample size was set at 85 participants per group. Therefore, a total of 170 participants will be enrolled in the study.

$k=n2/n1=1$

$n1=(\sigma21+\sigma22/K)(z1-\alpha/2+z1-\beta)2/\Delta2$

$n1= (302+302/1)(1.96+0.84)2/142$

$n1=72$

$n2=K\square n1=72$

(Reference: *Kim JB, Song WH, Park JS, Youn TJ, Park YH, Kim SJ, Ahn SG, Doh JH, Cho YH, Kim JW. A randomized, open-label, parallel, multi-center Phase IV study to compare the efficacy and safety of atorvastatin 10 and 20 mg in high-risk Asian patients with hypercholesterolemia. PLoS One. 2021 Jan 22;16(1):e0245481. doi: 10.1371/journal.pone.0245481. Erratum in: PLoS One. 2021 Oct 20;16(10):e0259072. PMID: 33481866; PMCID: PMC7822387*)

Statistical analyses:

A detailed Statistical Analysis Plan will be drawn up before breaking the randomisation code. We will analyse the data according to the intention-to-treat principle. Clinical outcome and laboratory outcome will be compared between the study groups by means of ordinal logistic regression, after adjustment for covariates.

We will perform pre-specified subgroup analyses of participants based on their risk as per the FRS, co-morbidities like DM, HTN, dose of standard care etc. Summary statistics by each treatment group will be tabulated at each visit. For continuous endpoints, the descriptive statistics include number of participants, mean, median, standard deviation, standard error, lower and upper quartiles, minimum, and maximum. For categorical endpoints, frequency, and percentage will be given. Missing data will not be imputed

Study withdrawal

Subjects have the right to withdraw from the study at any time and for any reason without prejudice to their future medical care by the physician or at the institution.

Withdrawal Criteria

Participation of a subject in this clinical study is entirely voluntary and may be discontinued for any of the following reasons:

- The subject withdraws consent or requests discontinuation from the study for any reason;
- Occurrence of any medical condition or circumstance that exposes the subject to substantial risk and/or does not allow the subject to adhere to the requirements of the protocol;
- Any serious adverse event (SAE), clinically significant adverse event, severe laboratory abnormality, or other medical condition which indicates to the Investigator that continued participation is not in the best interest of the subject;
- Pregnancy;
- Subject failure to comply with protocol requirements or study-related procedures
- Poor compliance
- Termination of the study by the Sponsor or the regulatory authority.

If a subject discontinues prematurely from the study due to the above criteria or any other reason, study staff should make every effort to document the clinical evaluations as far as possible in the electronic case report form (eCRF).

Withdrawn subjects may not be replaced. Withdrawal of consent for a study means that the subject does not wish to receive further protocol-required therapies or procedures, and the subject does not wish to or is unable to continue further study participation.

If a participant wishes to discontinue receiving the trial interventions, they will be encouraged to remain in the study for the purposes of data collection in line with the study schedule. If they wish to discontinue data collection, they will be asked to participate in an 'end of study' assessment at the time of withdrawal. Data collected prior to withdrawal will be used in the study analysis unless consent for this is specifically withdrawn. Should a decision to withdraw from the study be made, a reason for withdrawal will be sought but participants and/or carers can choose to withdraw without providing an explanation.

Trial conduct and practices/procedures:

Compliance with regulations and guidelines:

The trial will conform to the National Ethical Guidelines for Biomedical and Health Research Involving Human Participants (2017) ICMR and Good Clinical Practice guidelines for Clinical Trials in Ayurveda, Siddha and Unani Medicine (GCP-ASU), 2013.

Informed Consent:

Before a participant's participation in the clinical study, the investigator is responsible for obtaining written informed consent from the participant after adequate explanation of the aims, methods, anticipated benefits, and potential hazards of the study and before any protocol-specific screening procedures or any investigational product(s) is/are administered.

The acquisition of the informed consent and the participant's agreement or refusal is to be documented in the participant's medical records, and the informed consent is to be signed and personally dated by the participant and by the person who conducted the informed consent discussion. The original signed informed consent is to be retained in accordance with the CCRAS Research Policy, and a copy of the signed consent form is to be provided to the participant.
If a potential participant is illiterate or visually impaired and does not have a legally acceptable representative, the investigator must provide an impartial witness to read the informed consent to the participant and must allow for questions. Thereafter, both the participant and the witness must sign the informed consent to attest that informed consent was freely given and understood.

Institutional Ethics Committee:

A copy of the protocol, proposed informed consent, other written subject information, and any proposed advertising material must be submitted to the IEC for written approval. A copy of the written approval of the protocol and informed consent must be received by the CCRAS before recruitment of subjects into the study.
The investigator must submit and, where necessary, obtain approval from the IEC for all subsequent protocol amendments and changes to the informed consent. The investigator is to notify the IEC of deviations from the protocol or serious adverse events occurring at the site.

Study Documentation and Archive

The investigator is to maintain a list of appropriately qualified persons to whom he/she has delegated study duties. Source documents are original documents, data, and records from which the subject's CRF data are obtained. These include but are not limited to hospital records, clinical and office charts, laboratory and pharmacy records, diaries, and correspondence. CRF entries may be considered source data if the CRF is the site of the original recording (ie, there is no other written or electronic record of data).

The investigator and study staff are responsible for maintaining a comprehensive filing system of all study-related (essential) documentation, suitable for inspection at any time by representatives from CCRAS and/or applicable regulatory authorities.

The documents shall include:

- Subject files containing completed CRFs, informed consents, and subject identification list
- Study files containing the protocol with all amendments, Investigator's Brochure, , and all correspondence to and from the IEC and CCRAS

In addition, all original source documents supporting entries in the CRFs must be maintained and be readily available.

Data protection:

Personal identifiers will not be stored together with clinical information about the participant, but will be stored on a separate, password-protected computer with access only for persons in the Trial centre/ statistics unit who are responsible for data analysis. The code linking personal identifiers with clinical data will be destroyed 15 years after the publication of the primary report, of the trial.

Participant Confidentiality

The investigator must ensure that the participants confidentiality is maintained for documents submitted.

- Participants are to be identified by a unique identification number.

- For serious adverse events reported, participants are to be identified by their unique participant identification number, initials and date of birth (in accordance with local laws and regulations).

- Documents that are not submitted to (eg, signed informed consent s) are to be kept in confidence by the investigator, except as described below.

In compliance with the Good Clinical Practice (GCP) Guidelines, it is required that the investigator and institution permit authorized representatives of the sponsor, of the regulatory agency(s), and the IEC direct access to review the original medical records for verification of study-related procedures and data. Direct access includes examining, analysing, verifying, and reproducing any records and reports that are important to the evaluation of the study. The investigator is obligated to inform and obtain the consent of the participant to permit such individuals to have access to his/her study-related records, including personal information.

Study Monitoring and Data Collection

The CCRAS representative(s) and regulatory authority monitors can visit the investigator for the purpose of inspecting the facilities and,upon request, inspecting the various records of the clinical study (eg, CRFs and other pertinent data) provided that subject confidentiality is respected.

The Clinical Monitoring committee is responsible for verifying the CRFs at regular intervals throughout the study to verify adherence to the protocol; completeness, accuracy, and consistency of the data; and adherence to local regulations on the conduct of clinical research. The Clinical Monitoring committee is to have access to subject medical records and other study-related records needed to verify the entries on the CRFs. The investigator shall cooperate with the Clinical Monitoring committee to ensure that any problems detected in the course of these monitoring visits, including delays in completing CRFs, are resolved.

- Data capture for this study is planned to be in both hard copies of CRF and electronic format
- All source documentation supporting entries into the CRFs must be maintained and readily available
- To ensure the quality of clinical data across all subjects, clinical data management review is performed on subject data received at CCRAS. During this review, subject data are checked for consistency, omissions, and any apparent discrepancies. In addition, the data are reviewed for adherence to the protocol and GCP.

Investigator Responsibilities for Data Collection

The investigator is responsible for complying with the requirements for all assessments and data collection (including subjects not receiving protocol-required therapies) as stipulated in the protocol for each subject in the study. For subjects who withdraw prior to completion of all protocol-required visits and are unable or unwilling to continue the Schedule of Assessments, the reason for the same may be acquired telephonically to the participant or the care taker and enter the same in the CRF. The PI shall ensure that the data set(s) produced as an outcome of the study is/are as comprehensive as possible.

Handling of protocol violations and protocol amendments

The nature and reasons for the protocol violation shall be recorded in the CRF, in the source documents and in the monitoring visit report. In parallel, corrective and/or preventive actions will be undertaken and documented, including any retraining of the investigator and site staff. All participants who have been included in the trial will be followed up, irrespective of whether treatment was discontinued prematurely, or whether the protocol was violated.

All important changes to the trial will be specified in protocol amendments. Amendments must be approved by the Sponsor, and the PI has the responsibility to seek approval from the competent authorities the ethics committees. The approval shall also be conveyed to the sponsor

Safety data collection, recording, and reporting:

Clinical trials provide the evidentiary basis for regulatory approvals of safe and effective interventions. The safety of the trial intervention, will be evaluated by examining the occurrence of all adverse events and serious adverse events during the course of the study.

Definitions

Adverse event (AE): Any untoward medical occurrence in a participant to whom a study intervention or procedure has been administered, including occurrences which are not necessarily caused by or related to that intervention. An AE, therefore, does not necessarily have a causal relationship with the treatment. In this context, "treatment" includes all interventions (including comparative agents) administered during the course of the study. The definition of adverse events includes worsening of a pre-existing medical condition. Worsening indicates that the pre-existing medical condition or underlying disease (eg, diabetes, migraine headaches,) has increased in severity, frequency, and/or duration more than would be expected, and/or has an association with a significantly worse outcome than expected.

Serious Adverse Events

A serious adverse event is defined as an adverse event that meets at least 1 of the following serious criteria

- Results in death
- Is life-threatening (refers to an event in which the subject was at risk of death at the time of the event; it does not refer to an event which hypothetically might have caused death if it were more severe)
- Requires hospitalisation, or prolongation of existing hospitalisation
- Results in persistent or significant disability or incapacity
- Consists of a congenital anomaly or birth defect
- Is otherwise considered medically significant by the investigator

Causality: The assignment of the causality should be made by the investigator responsible for the care of the participant using the definitions in the table below. All adverse events judged as having a reasonable suspected causal relationship to a study procedure (i.e definitely, probably or possibly related) are considered to be related adverse events.

**Relationship**	**Description**
Unrelated	There is no evidence of any causal relationship
Unlikely	There is little evidence to suggest there is a causal relationship (e.g. the event did not occur within a reasonable time after administration of the study procedure). There is another reasonable explanation for the event (e.g. the participant's clinical condition, other concomitant treatment).
Possible	There is some evidence to suggest a causal relationship (e.g. because the event occurs within a reasonable time after administration of the study procedure). However, the influence of other factors may have contributed to the event (e.g. the participant's clinical condition, other concomitant treatments).
Probable	There is evidence to suggest a causal relationship and the influence of other factors is unlikely.
Definitely	There is clear evidence to suggest a causal relationship and other possible contributing factors can be ruled out.
Not assessable	There is insufficient or incomplete evidence to make a clinical judgement of the causal relationship.

Recording and reporting of serious adverse events

Serious adverse events will be recorded in the case report forms. In case of unexpected serious adverse events, the CCRAS Headquarters should be notified immediately and within 24 hours at the latest.

Reports of suspected unexpected serious adverse reactions (SUSARs), with all relevant information, will be reported in an expedited manner by the Sponsor, to the competent authority, the ethics committee, and the Data Monitoring Committee.

The investigator must assign the following adverse event attributes:

- Adverse event diagnosis or syndrome(s), if known (if not known, signs or symptoms),
- Dates of onset and resolution (if resolved),
- Severity
- Assessment of relatedness to any study-mandated activity or procedure, and
- Action taken.

Interim Analysis

An Interim analysis, if required would be done when at least 50% participants have completed their 3 months of therapeutic regime.

Co-ordination of trial

Co-ordinating Centre:

Central Council for Research in Ayurvedic Sciences (CCRAS),
Ministry of Ayush,
Government of India, Jawahar Lal Nehru Bhartiya Chikitsa Evam Homoeopathy Anusandhan Bhawan, 61-65,Institutional Area, Opposite 'D' Block, Janakpuri, New Delhi-110058,
India

Trial Monitoring

CCRAS's Biostatistical Monitoring Unit) and the technical officers those are directly involved in this project will monitor the progress of the trial through regular site visits. The purpose of these visits would be to ensure strict adherence to the trial protocol, correct completion of the forms and to discuss any problems being faced by the research staff at the participating site.

Training of project personnel

The Principal Investigator / Project Co-ordinator have to ensure standardized methods of data collection, if required, and would train the Investigating physicians and the paramedical staff involved in the project.

Laboratory Examination: (BT & AT)

- **Haematology**
  - Haemoglobin : ________________ g/dl
  - T.L.C. : ________________ / cu.mm.
  - D.L.C. : N ____ % E ____ % B ____ % L ____ % M _____ %
  - E.S.R. : _________ mm (at the end of 1st hour)
  - Blood Sugar :Fasting ___________ mg%
  - HbA1c: __________________ %
- **Bio-chemistry:**
  - Blood Urea : ________________ mg/dL
  - Serum Uric Acid: ________________ mg/dl.
  - Serum Creatinine : ________________ mg/dL
  - S.G.O.T.(A.S.T.) : ________________ IU/L
  - S.G.P.T. (A.L.T.) : ___________ IU/L
  - Total protein: ___________ gm/dl
  - S.Albumin: ___________ gm/dl
  - S.Globulin: ___________ gm/dl
  - A/G ratio: ___________
  - Serum Bilirubin:
    - Conjugated bilirubin _______ mg/dl
    - Unconjugated bilirubin ______ mg/dl
  - Serum Alkaline Phosphatase: _______ IU/L
  - Apolipoprotein
  - Adiponectin
  - Leptin
  - Ghrelin

Stool

Gut Microbiome:

Urine:

Routine

Microscopy

Other Investigations

ECG: At screening and 84th day

Budget requirement:

Head wise requirement along with justification

**Particulars**	**1st year**	**2nd year**	**Total**	**Justifications**
Publication in peer reviewed journals	0	300000	300000	Protocol publication in the first year and study publication after statistical analysis<br>Publication/open access charges
SRF (Ayurveda) (35,000+18% HRA= @41,300/- pm)	495600	495600	991200	Assisting the PI with screening, data documentation in CRF & e-format etc
Lab Technician @16,000/pm	192000	192000	384000	For stool sample collection, blood sample collection, storage etc.
Trial drug (Atorvastatin 20mg/40mg)	200000	200000	400000	To procure trial drugs of standard care (By the AIIMS team) @ Rs.250/10 tab approx.<br>For 170 patients= 170 x 90 tab (Rs.2250/-)= Rs.382500/-<br>Rounded off to 4 lakh
**Laboratory Investigation** (ECG ,TFT, KFT, LFT, LIPID profile, FBS, HBA1C, Hs-CRP, CBC, Grehlin, Leptin, TNF-Alpha, Adiponectin, Apolipoprotein A1 Apolipoprotein B, IL-6, Insulin, Gut microbiota)*	5287800	0	5287800	To be performed at AIIMS Biochemistry/Central lab. and one test to be outsourced (Cost X Times X No. Of Subjects)
Reagents & solutions for preservation of stool sample, vials & tubes etc. for sample collection.	100000	100000	200000	Reagents & solutions for preservation of stool sample (eg: modified Cary Blair medium), Vials & syringes, EDTA tubes for Blood collection, aliquots, stool collection pan, sample storage equipment, sample transport

				accessories are required
**Stationary**	150000	100000	250000	For purchase of files, paper, register, computer cartridge, printing of CRF
**Travelling support/incidental support expenses for research subject**	85000	0	85000	To be paid to research subject on each visits for 5 visits. (100 x 5 visits x 170 subjects)
**Local TA/conveyance for investigator/Honoraria for IEC member including conveyance**	50000	50000	100000	For local TA of project investigators, personnel to camp sites, TA/conveyance for investigator/Honoraria for IEC member including conveyance
**Contingency or Miscellaneous expenses**	100000	50000	150000	To meet unforeseen expenses
**Total**	6660400	1487600	8148000	
**Overhead Institutional charges 5%**	333020	74380	407400	
**Grand Total**	6993420	1561980	8555400	
**Grand Total**	**Rs.85,55,400/- (Rupees Eighty five lakh fifty five thousand four hundred only)**			

***Justification for Laboratory investigation:**

**Investigation**	**Rate/ test (INR)**	**No of times test to be done**	**Amount for 170 participants**	**Amount for screen failure@20% (for screening tests only) (24)**	**Total**
Lipid Profile	125	5 times (Screening,28,56,84,112)	127500	5100	132600
CBC	15	2 times (screening, 84th day)	7440	744	8184
FBS	50	2 times (screening, 84th day)	20400	2040	22440

HbA1C	150	2 times (screening, 84th day)	61200	6120	67320
LFT	220	2 times (screening, 84th day)	89760	8976	98736
RFT	400	2 times (screening, 90th day)	163200	16320	179520
TSH	250	Screening	51000	10200	61200
ECG	Free	Screening, 84th day	0	0	0
Apolipoprotein A1	Rs.46000/- for 85 tests	2 times (baseline, 84th day)	220800	0	220800
Apolipoprotein B	Rs.46000/- for 85 tests	2 times (baseline, 84th day)	220800	0	220800
Adiponectin	Rs.34000/- for 85 tests	2 times (baseline, 84th day)	163200	0	163200
leptin	Rs.54000/- for 85 tests	2 times (baseline, 84th day)	259200	0	259200
Ghrelin	Rs.46000/- for 85 tests	2 times (baseline, 84th day)	220800	0	220800
Hs-CRP	25	2 times (baseline, 84th day)	10200	0	10200
IL-6	150	2 times (baseline, 84th day)	61200	0	61200
TNF Alpha	Rs.36000/- for 85 tests	2 times (baseline, 84th day)	172800	0	172800
Gut Microbiome	8000	2 times (baseline, 84th day)	3264000	0	3264000
INSULIN	Rs.26000/- for 85 tests	2 times (baseline, 84th day)	124800	0	124800
Total			5238300	49500	5287800

Total	Rs 5287800/-

"Efficacy and safety of Ayurveda Formulation 'Trikatu' as add-on to standard care in Dyslipidemia- A Prospective Randomized Double Blind Placebo Controlled Trial"

Consent Form - to be signed on the day of Screening

Centre Code: ---------------------

I certify that I have disclosed all details about the study in the terms easily understood by the subject.

Date: ____________________________

Signature of the investigator:______________________

Name:____

CONSENT BY PARTICIPANT:

1. I confirm that I have read / the study has been explained to me adequately and I have understood the information sheet for the above study and had the opportunity to ask questions.
2. I hope to complete the study, but I understand that my participation is voluntary and that I am free to withdraw at any time, without giving a reason, and without my medical care or legal rights being affected.
3. I understand that my doctor will provide information about my progress, in confidence, to the related research personnel and sponsor.
4. I understand that the information held by the Investigators and researchers and records maintained for the study might be used to follow up on my health status.
5. I understand that the information will be used for medical research only and that I will not be identified in any way in the analysis and reporting of the results. I understand that sections of any of my medical notes may be looked at by the Sponsors or responsible individuals from the members of the Institutional Ethics Committee (IEC), Regulatory authorities or Court, if necessary. I give permission for these individuals to have access to my records.
6. I understand what is involved in this trial and agree to take part in the clinical trial entitled **"Efficacy and safety of Ayurveda Formulation 'Trikatu' as add-on to standard care in Dyslipidemia- A Prospective Randomized Double Blind Placebo Controlled Trial"** for a period of 16 weeks (including the follow up period of 4 weeks).

Date: ________________

Name of participant ______________________

Signature or Thumb impression _________

Date: ________________

Name of witness: ______________________

Signature or Thumb impression: _________

Relationship: ________________________

(Note: - Three copies of the consent form will be made)

PATIENT INFORMATION SHEET (PIS) & CONSENT FORM

Invitation Paragraph: We are inviting you to take part in a study to observe whether it is possible to manage elevated cholesterol levels or impaired cholesterol parameters (known as dyslipidemia) by using an Ayurvedic formulation viz., Trikatu Tablet along with the standard treatment for dyslipidemia. Please read this information sheet carefully before deciding whether to take part.

1. **Study title:**

"*Efficacy and safety of Ayurveda Formulation 'Trikatu' as add-on to standard care in Dyslipidemia- A Prospective Randomized Double Blind Placebo Controlled Trial*".

2. **Why have I been invited to take part in this study?**

You are being invited to take part in this research study because you are diagnosed as having dyslipidemia and fits the selection criteria for the study. This research study would recruit a total of 170 participants with dyslipidemia for 2 years.

3. **What is the purpose of the study?**

This is a research study being conducted by the Central Council for Research in Ayurvedic Sciences (CCRAS), Ministry of AYUSH, Government of India, to explore the role of a time-tested Ayurvedic formulation, "Trikatu' in tablet form to effectively manage dyslipidemia..

4. **Do I have to take part?**

No, your participation in the study is completely voluntary and you will not be at any disadvantage if you decide not to take part. If you do decide to take part you will be given this information sheet to keep and be asked to sign a consent form. If you agree to take part you are still free to withdraw at any time and without giving any reason. This will not affect the standard of care you receive.

5. **What will happen to me if I take part?**

If you agree to take part in this study you will be prescribed an Ayurveda Formulation **'Trikatu'** in tablet form or a placebo for 84 days along with the standard treatment for dyslipidemia, Atorvastatin as per the clinical assessment done by your physician. A placebo is a substance with no therapeutic effect and appears similar to the trial drug. This study is a randomized double blind trial, in which neither your physician nor you would be aware whether you are receiving

the medicine or placebo. You will have a 50% chance to be in either of the two groups as the allocation of the participant is done through computer generated randomization process. However, irrespective of whether you receive the Ayurveda trial intervention or placebo, you will receive the standard care as per the severity of your condition as adjudicated by your physician.

You have to come for follow-up clinical examination & Investigations on the 28th, 56th, 84th, and 112th days. In the first visit i.e. on baseline (Day 1) you may have to devote approximately 2 hours to facilitate your investigating physician in recording your medical history in detail and to perform the clinical examination in detail. Thereafter, in the subsequent visits, it may take nearly 15 – 20 minutes to make the assessment. During the screening, and the follow up visits, blood samples in the range of 10-20 ml would be collected for laboratory testing. It is also required that you provide a stool sample at the baseline and 84th day follow-up, You will be nominally compensated for your loss of wages/conveyance charges by paying an amount of Rs.100 (One hundred only) for every visit to the hospital during the study period i.e. Baseline, Day 28, Day 56, Day 84 (AT) & Day 112 (follow up visit) (An amount of Maximum Rs. 500 / - [Five hundred rupees only] for Total 05 visits).

6. What do I have to do?

You have to adhere to the instructions given to you by your Investigating physician regarding taking the medicines as advised and reporting for follow-up on the prescribed day. During the trial, you can safely continue with your regular medications (if any, after informing your physician of the same) and the only word of caution is that you should follow and obey the instructions of your investigator very religiously while participating in the study.

7. What is the drug or procedure that is being tested?

The participants selected in the clinical trial will be given the Ayurvedic Formulation Trikatu Tablet or its matching placebo in the dose as given below:

Group I: Tablet Trikatu 1000 mg twice daily with lukewarm water one hr after food for 84 days

Group II: Matching placebo 1000 mg twice daily with Luke warm water one hr after food 84 days

Your standard care includes Atorvastatin in the dose of 20 mg/40 mg as per the contemporary guidelines for the management of dyslipidemia, which will be given to you by your physician.

8. What are the alternatives for diagnosis or treatment?

The contemporary management of Dyslipidaemia involve the use of lipid-modifying drugs which are effective in bringing the lipid levels to goal thereby reducing the risk of CV events. The therapy suitable for you will be decided by the ASCVD risk assessment, wherein you would be prescribed, statins in moderate or high intensity or other anti-dyslipidemic medicines. If you are in low risk for cardiovascular diseases, you would be prescribed lifestyle interventions and patients with intolerance to statins would receive other pharmacotherapy.

9. What are the possible disadvantages and risks of taking part?

The Ayurvedic Formulation, Trikatu, has been in use for a long time, yet individual-specific side effects may appear at any time during the clinical trial, which you have to report immediately to your investigating physician. You may feel acidity, bloating or gastric upset following the use of trial interventions and the same shall be intimated to the study personnel.

Even though its use is not associated with any known side effects, pregnant and lactating women are excluded from the trial. Any blood samples collected will be in line with best practice guidelines to minimize any side effects.

'12. What are the possible benefits of taking part?

It is not guaranteed that you will get cured of **Dyslipidaemia** after completing the course of the trial but your participation will help us in generating sufficient data to validate the efficacy and safety of these therapies in managing/treating this condition. Since you are not deprived of the standard care, you will get the benefit of managing your condition.

You would be monitored closely and would benefit from the close monitoring and you would avail a multitude of investigations which would normally not be done in clinical scenario and this would help you assess your metabolic status and do the needful for primary prevention against metabolic or cardiovascular diseases.

13. What if new information becomes available?

If during the clinical trial, some new information becomes available about the Ayurvedic treatment being studied, you will be informed about that by your investigating physician after

which you are free to decide whether you want to continue in the study or not. If you decide to withdraw, this will not at all affect your routine care in the hospital. If you decide to continue in the study, you will be asked to sign a fresh consent form. On the other hand, upon receiving any new information, your investigating physician might consider it to be in your best interests to withdraw you from the study. Your investigating physician will explain the reasons for dropping you from the study and arrange for your routine care to continue.

14. What happens when the research study stops?

You will be given appropriate advice for a future line of treatment.

15. What if something goes wrong?

If something goes wrong then please contact the research team as soon as possible to explain the problem. We will work with you to find a resolution .Compensation for any adverse effect caused by taking part in this study and related to the study would be taken care of by appropriate treatment or referrals.

Contact address of the Principal Investigator/ Co-investigator:-

Principal Investigator
Dr.Kshirod Kumar Ratha,
Research Officer (Ayurveda)
Mob:9874612213
Central Ayurveda Research Institute for Hepatobiliary disorders. Bhubaneswar, Odisha

Co-investigator
Dr. Suchanda Sahu
Additional Professor,
Department of Biochemistry, AIIMS, Siju
Bhubaneswar
Mob:9438884031,

Dr. Meda Mruthyumjaya Rao
Director
Central Ayurveda Research Institute for Hepatobiliary disorders. Bhubaneswar, Odisha
Telephone/ Mob.: 9040224463(M)

Dr. Sujata Devi
Assoc. Prof., General Medicine
Postal address: AIIMS, Sijua, Bhubaneswar
Mob: 9438884203

16. Will my taking part in this study be kept confidential?

Yes, all your information will be kept confidential but any of your medical records may be inspected by the sponsors to analyse the results. They may also be looked at by members of the Institutional Ethics Committee and by Regulatory authorities / Court to check that the study is being carried out correctly. Your name, however, will not be made public and any sensitive matter regarding your state of health will be kept confidential.

17. What if I want to withdraw from the study?

If, at any stage you wish to leave the study, then you can. There is no problem should you wish to stop taking part and it is entirely up to you. There will be no disadvantage to you if you withdraw. If you lose capacity to consent during your participation in the study, you will be withdrawn from the study. Identifiable data already collected with your consent may be retained and used in the study.

18. What will happen to the results of the research study?

The results of the clinical trial will be published in leading medical journals so that other doctors and researchers can benefit from the results. You can ask your investigating physician for a copy of the publication. If published, your identity and personal details will be kept strictly confidential. No named information about you will be published in any of the trial reports.

18. Who is organizing and funding the research?

Central Council for Research in Ayurvedic Sciences (CCRAS), New Delhi is organizing and sponsoring the research.

19. Who has reviewed the study?

Institutional Ethics Committee(s) of AIIMS Bhubaneswar has reviewed the study

20. Contacts for Further Information

If desirous of any relevant information at any stage of the clinical trial, you may feel free to ask your investigating physician for that information. You would be given a copy of the information sheet and a signed consent form.

Contact address and telephone no. of the Investigator -.

Principal Investigator
Dr.Kshirod Kumar Ratha,
Research Officer (Ayurveda)
Mob:9874612213
Central Ayurveda Research Institute for Hepatobiliary disorders. Bhubaneswar, Odisha

Co-investigator
Dr. Suchanda Sahu
Additional Professor,
Department of Biochemistry, AIIMS, Siju
Bhubaneswar
Mob:9438884031,

Dr. Meda Mruthyumjaya Rao
Director
Central Ayurveda Research Institute for Hepatobiliary disorders. Bhubaneswar, Odisha
Telephone/ Mob.: 9040224463(M)

Dr. Sujata Devi
Assoc. Prof., General Medicine
Postal address: AIIMS, Sijua, Bhubaneswar
Mob: 9438884203

Translation of Patient information sheet into regional language to be done by Investigator

INSTITUTIONAL ETHICS COMMITTEE

(Registration No. ECR/534/Inst/OD/2014/RR-20)

All India Institute of Medical Sciences Bhubaneswar

Level 3 Academic Block, AIIMS Bhubaneswar (At Sijua)
Bhubaneswar 751019, Odisha
Email: iec@aiimsbhubaneswar.edu.in **Phone:** 0674-2476083

Ref Number: T/EMF/Biochem/22/15 **Date: June 09, 2022**

Chairperson

Dr Suresh Chandra Dash

Members

Ms Swarna Misra
(Lay Person)

Dr Navaneeta Rath
(Social Scientist)

Mr. Santanu K Sarangi
(Legal Person)

Dr Subash Chandra Samal
(Clinician)

Dr Rituparna Maiti
(Pharmacologist)

Dr Srujana Mohanty
(Basic Scientist)

Dr Sweta Singh
(Clinician)

Dr Santosh Kumar Mahallik
(Clinician)

Dr Balamurugan Ramadass
(Basic Scientist)

Dr Trupti Swain
(Pharmacologist)

Dr Priti Das
(Pharmacologist)

Dr Sonali Kar
(Scientific Member)

Member- Secretary

Dr Arvind Kumar Singh

Dr Suchanda Sahu
Additional Professor,
Department of Biochemistry
AIIMS, Bhubaneswar

Subject: "Efficacy and safety of Ayurveda Formulation 'Trikatu' as add-on to standard care in Dyslipidemia- A Randomized Controlled Trial."

Dear Dr Suchanda Sahu,

This is regarding your above-mentioned project proposal which was discussed in the Institutional Ethics Committee, AIIMS Bhubaneswar meeting held on May 14, 2022 (Saturday) and your subsequent letter dated June 08, 2022 responding to queries raised during IEC meeting.

The study is approved from ethical angle prospectively with effect from **June 09, 2022** till the entire period of the conduct of study according to the study duration mentioned in the protocol under direct responsibility of Dr Suchanda Sahu, Principal Investigator.

As a Principal Investigator, you are responsible for following requirements as applicable for the present protocol.

1. All co-investigators must be kept informed of the status of the project.
2. No significant change to the protocol should be made and implemented without prior intimation and approval of the IEC
3. IEC should be reported about all Serious Adverse Events (SAEs) occurring during the study.
4. Only approved informed consent form and participant information sheet to be used for enrolment of the participants. All consent forms and other documents must be archived safely with PI for IEC audit
5. A six-monthly study progress report of the project must be submitted to IEC
6. It is hereby confirmed that neither you nor any of the study team members have participated in the voting/ decision making process of Institute Ethics Committee of AIIMS Bhubaneswar related to this study.

Chairperson
(IEC AIIMS Bhubaneswar)
अध्यक्ष / Chairperson
संस्थागत आचार समिति
Institutional Ethics Committee
एम्स, भुवनेश्वर / AIIMS,Bhubaneswar
